# Atherosclerosis and liver inflammation induced by increased dietary cholesterol intake: a combined transcriptomics and metabolomics analysis

**DOI:** 10.1186/gb-2007-8-9-r200

**Published:** 2007-09-24

**Authors:** Robert Kleemann, Lars Verschuren, Marjan J van Erk, Yuri Nikolsky, Nicole HP Cnubben, Elwin R Verheij, Age K Smilde, Henk FJ Hendriks, Susanne Zadelaar, Graham J Smith, Valery Kaznacheev, Tatiana Nikolskaya, Anton Melnikov, Eva Hurt-Camejo, Jan van der Greef, Ben van Ommen, Teake Kooistra

**Affiliations:** 1 Department of Vascular and Metabolic Diseases, TNO-Quality of Life, BioSciences, Gaubius Laboratory, Zernikedreef 9, 2333 CK Leiden, The Netherlands; 2 Department of Vascular Surgery, Leiden University Medical Center, Albinusdreef 2, 2300 RC Leiden, The Netherlands; 3 Department of Physiological Genomics, TNO-Quality of Life, BioSciences, Utrechtseweg 48, 3704 HE Zeist, The Netherlands; 4 GeneGo Inc., Renaissance Drive, St Joseph, MI 49085, USA; 5 Department of Analytical Research, TNO-Quality of Life, Quality and Safety, Utrechtseweg 48, 3704 HE Zeist, The Netherlands; 6 AstraZeneca, CV&GI Research, Silk Road Business Park, Macclesfield, Cheshire SK10 2NA, UK; 7 Vavilov Institute for General Genetics, Russian Academy of Science, Gubkin Street 3, 117809 Moscow, Russia; 8 AstraZeneca CV&GI Research, 43183 Mölndal, Sweden

## Abstract

**Background:**

Increased dietary cholesterol intake is associated with atherosclerosis. Atherosclerosis development requires a lipid and an inflammatory component. It is unclear where and how the inflammatory component develops. To assess the role of the liver in the evolution of inflammation, we treated ApoE*3Leiden mice with cholesterol-free (Con), low (LC; 0.25%) and high (HC; 1%) cholesterol diets, scored early atherosclerosis and profiled the (patho)physiological state of the liver using novel whole-genome and metabolome technologies.

**Results:**

Whereas the Con diet did not induce early atherosclerosis, the LC diet did so but only mildly, and the HC diet induced it very strongly. With increasing dietary cholesterol intake, the liver switches from a resilient, adaptive state to an inflammatory, pro-atherosclerotic state. The liver absorbs moderate cholesterol stress (LC) mainly by adjusting metabolic and transport processes. This hepatic resilience is predominantly controlled by SREBP-1/-2, SP-1, RXR and PPARα. A further increase of dietary cholesterol stress (HC) additionally induces pro-inflammatory gene expression, including pro-atherosclerotic candidate genes. These HC-evoked changes occur via specific pro-inflammatory pathways involving specific transcriptional master regulators, some of which are established, others newly identified. Notably, several of these regulators control both lipid metabolism and inflammation, and thereby link the two processes.

**Conclusion:**

With increasing dietary cholesterol intake the liver switches from a mainly resilient (LC) to a predominantly inflammatory (HC) state, which is associated with early lesion formation. Newly developed, functional systems biology tools allowed the identification of novel regulatory pathways and transcriptional regulators controlling both lipid metabolism and inflammatory responses, thereby providing a rationale for an interrelationship between the two processes.

## Abstract 2

With increasing dietary cholesterol intake the liver switches from a mainly resilient to a predominantly inflammatory state, which is associated with early lesion formation.

## Background

Atherosclerosis is a multifactorial disease of the large arteries and the leading cause of morbidity and mortality in industrialized countries [1]. There is ample evidence that hypercholesterolemia (that is, elevated plasma levels of low-density lipoprotein (LDL) and very low-density lipoprotein (VLDL)) induced by genetic modification or enhanced intake of dietary lipids is a major causative factor in atherogenesis [2,3]. It is equally clear that from the very beginning of lesion formation, atherogenesis requires an inflammatory component, which is thought to drive the progression of the disease [4,5]. Indeed, some of the variation in the rate of lesion progression in different individuals may relate to variations in their basal inflammatory state [6,7]. However, while the inflammatory processes in the complex evolution of the lesion from the early fatty streak to a fibrous plaque are considered self-perpetuating phenomena, the initial trigger and origin of the inflammatory component in hypercholesterolemia remains enigmatic [6,8].

Recent observations by us and others suggest that the liver plays a key role in the inflammatory response evoked by dietary constituents (reviewed in [8,9]). For example, liver-derived inflammation markers such as C-reactive protein (CRP) and serum amyloid A (SAA) increase rapidly (within days) after consumption of an excess amount of dietary lipids [8,10], and thus by far precede the onset of early aortic lesion formation [8]. These findings suggest that nutritional cholesterol itself may contribute to the evolution of the inflammatory component of atherogenesis. We postulate that pro-atherogenic inflammatory factors originate at least partly from the liver. We also hypothesize that these factors come into play at high dietary cholesterol doses because of the exponential rather than linear nature of the relationship between cholesterol intake (measured as cholesterol plasma levels) and atherosclerotic lesion size [11,12] as specified in more detail in Additional data file 1.

In this study we sought evidence for the hypothesis that inflammation and hypercholesterolemia are not separate factors, but closely related features of the same trigger, dietary cholesterol. In particular, using a variety of newly developed functional bioinformatics tools, we addressed the question of how the liver responds to increasing dietary cholesterol loads at the gene transcription level and analyzed how hepatic cholesterol metabolism is linked to the hepatic inflammatory response, including underlying regulatory mechanisms. Notably, all analyses were performed at a very early stage of the atherogenic process (that is, after 10 weeks of cholesterol feeding) to limit potential feedback reactions from the vessel wall.

An established model for cholesterol-induced atherosclerosis, ApoE*3Leiden transgenic (E3L) mice, allowed the application of experimental conditions that mimic the human situation: E3L mice display a lipoprotein profile similar to that of humans suffering from dysbetalipoproteinemia and develop atherosclerotic lesions that resemble human lesions with regard to morphology and cellular composition [13,14]. E3L mice were exposed to increasing doses of dietary cholesterol (as the only dietary variable modulated), and liver genome and metabolome datasets were analyzed in a unique context, that is, at the time point of first lesion development. Advanced (functional) bioinformatical analysis allowed us to merge metabolome and transcriptome datasets and to analyze pathways and biochemical processes comprehensively. Recent advances in systems biology (for example, new biological process software for network building and data mining) have enabled us to discover significant relationships and to identify transcriptional master regulators that control gene alteration and are ultimately responsible for effects at the process level.

## Results

### Effect of dietary cholesterol load on plasma lipids and early atherosclerosis

**Figure 1:**
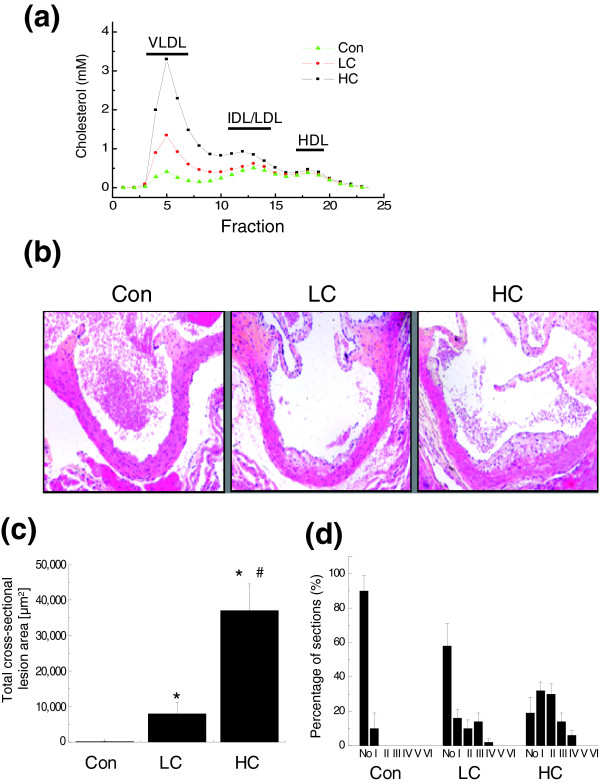
Analysis of plasma lipids and atherosclerosis. **(a) **Lipoprotein profiles of the Con, LC and HC groups at ten weeks. **(b) **Representative photomicrographs of cross-sections of the aortic root area. **(c) **Total cross-sectional lesion area in the aortic root. **(d) **Lesion severity of the treatment groups determined according to the lesion classification of the American Heart Association (I-VI). Data are presented as means ± standard deviation. *P < 0.05 versus Con; ^#^*P *< 0.05 versus LC.

Treatment of female E3L mice with a cholesterol-free (Con), a low-cholesterol (LC) or a high-cholesterol (HC) diet resulted in total plasma cholesterol concentrations that stabilized at 5.9 ± 0.3 mM, 13.3 ± 1.9 mM and 17.9 ± 2.4 mM, respectively. The increase in plasma cholesterol in the LC and HC groups was confined to the pro-atherogenic lipoprotein particles VLDL and LDL (Figure 1a). High-density lipoprotein (HDL) levels and plasma triglyceride levels were comparable between the groups (Figure 1a and Table 1). The plasma levels of alanine aminotransferase (ALAT) and aspartate aminotransferase (ASAT), two markers of liver function, were comparable in the Con and LC groups and slightly elevated in the HC group.

**Table 1:** Effects of dietary cholesterol on plasma lipids and inflammation markers

	Con	LC	HC
Body weight (start) (g)	20.3 ± 1.5	20.8 ± 1.5	20.6 ± 0.9
Body weight gain (g)	0.4 ± 0.7	0.7 ± 0.8	0.6 ± 0.5
Food intake (g/day)	2.6 ± 0.2	2.9 ± 0.3*	2.5 ± 0.2^†^
Plasma cholesterol (mM)	5.9 ± 0.3	13.3 ± 1.9*	17.9 ± 2.4*^†^
Plasma triglyceride (mM)	1.7 ± 0.4	2.3 ± 0.3	2.1 ± 0.7
Plasma E-selectin (μg/ml)	44.3 ± 2.3	44.3 ± 6.3	55.1 ± 8.5*^†^
Plasma SAA (μg/ml)	2.8 ± 0.6	4.7 ± 1.7	8.3 ± 2.7*^†^
Plasma ALAT (U/mL)	48 ± 44	45 ± 22	75 ± 23
Plasma ASAT (U/mL)	260 ± 123	237 ± 57	569 ± 221*^†^

Three groups of female E3L mice were fed either a cholesterol-free (Con) diet or the same diet supplemented with 0.25% (LC) or 1.0% (HC) w/w cholesterol. Listed are the average body weight at the start (t = 0) of the experimental period together with the body weight gain, the average daily food intake and the average plasma levels of cholesterol, triglycerides, E-selectin, serum amyloid A (SAA), alanine aminotransferase (ALAT) and aspartate aminotransferase (ASAT). All data are mean ± standard deviation. *P < 0.05 versus Con; ^†^*P *< 0.05 versus LC (ANOVA, least significant difference *post hoc *test).

After 10 weeks of dietary treatment, the animals were euthanized to score early atherosclerosis. Longitudinally opened aortas of the Con and LC groups were essentially lesion-free (*en face *oil red O-staining), while aortas of the HC group already contained lesions (not shown). Consistent with the presence of atherosclerosis in the HC group, the vascular inflammation marker E-selectin was elevated only in this group (Table 1). Early onset of atherosclerosis was analyzed in more detail in the valve area of the aortic root (Figure 1b), a region in which lesions develop first [15]. The total cross-sectional lesion area under basal conditions was 1,900 ± 900 μm^2 ^(Con group; Figure 1c). Compared to the Con group, the lesion area was relatively moderately increased in the LC group (4.2-fold; *P *< 0.05) and strongly increased in HC (19.5-fold; *P *< 0.05), confirming the exponential rather than linear relationship between total plasma cholesterol levels and the lesion area.

Next, lesions in the aortic root were graded according to the classification of the American Heart Association. Under control conditions (Con); only about 10% of the aortic segments contained lesions, all of which were very mild type I lesions not identified by *en face *staining (Figure 1d). In the LC group, more (40%) aortic segments showed lesions, of which 38% were mild type I-III lesions and 2% were severe type IV lesions. In the HC group, 81% of the aortic segments displayed lesions, most of which were mild lesions (76% type I-III lesions; 5% type IV). The predominance of mild-type lesions confirms an early stage of atherosclerotic disease in all groups. Notably, a positive association was observed between the cross-sectional lesion area and the plasma levels of SAA, an inflammation marker formed in liver. SAA was significantly elevated in the HC group, pointing to a hepatic inflammatory response to cholesterol feeding (Table 1) that is associated with early atherosclerotic lesion formation.

### Analysis of the hepatic gene response to increasing doses of dietary cholesterol

**Figure 2:**
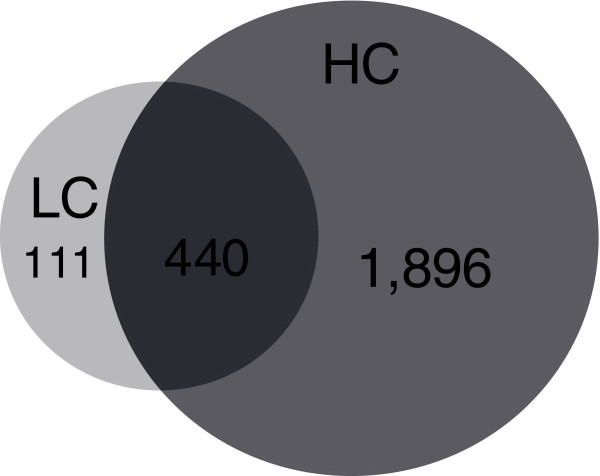
Venn diagram of significantly differentially expressed genes in the LC and HC groups compared to the Con group. ANOVA *P *< 0.01 and FDR (predicted) <0.05 resulted in 2,846 probesets, and subsequent *t*-tests with *P *< 0.01 for HC versus Con and/or LC versus Con resulted in the 2,447 probesets shown.

To get insight into the complex traits underlying the (patho)physiological response of the liver to dietary cholesterol, whole-genome and metabolome measurements were made. Compared to the Con group, a relatively small number of genes (551) significantly changed with LC treatment (Figure 2). HC treatment modulated most (440 out of 551) of these genes and, additionally, affected 1,896 other genes. The individual gene expression profiles within a treatment group were very similar and formed clusters as confirmed by hierarchical clustering analysis (not shown). Differences in gene expression between the treatment groups were validated and confirmed for a selected group of genes by RT-PCR (Additional data file 2).

Standard Gene Ontology (GO) biological process annotation allowed categorization of 52% of the differentially expressed genes based on their biological function (Table 2). LC treatment predominantly affected genes belonging to lipid and lipoprotein metabolism, protein metabolism, carbohydrate metabolism, energy metabolism and transport. HC treatment affected the same GO groups but, additionally, also genes relevant to immune and inflammatory responses, cell proliferation, apoptosis, cell adhesion and cytoskeleton integrity (Table 2 and Additional data file 3).

**Table 2:** Overview of genes that are differentially expressed in response to cholesterol

	LC			HC		
		
GO category	Up	Down	Total	Up	Down	Total
Lipid and lipoprotein metabolism (includes cholesterol and steroid metabolism)	8	50	58	37	114	151
Protein metabolism (includes protein folding and breakdown)	34	14	48	143	98	241
Other metabolism (includes carbohydrate metabolism)	32	19	51	122	130	252
Generation of precursor metabolites and energy	10	15	25	24	47	71
Transport	31	15	46	125	77	202
Immune and stress response/inflammation	19	7	26	99	49	148
Cell proliferation/apoptosis	9	3	12	37	18	55
Cell adhesion/cytoskeleton	10	1	11	76	8	84

Differentially expressed genes of LC and HC groups (ANOVA FDR < 0.05 and *t*-test compared to Con group *P *< 0.01) were analyzed according to standard GO biological process annotation and grouped into functional categories.

To refine the liver transcriptome data analysis and to define which biological processes are switched on/off with increasing dietary cholesterol loads, we performed gene enrichment analysis in four different functional ontologies: biological processes, canonical pathway maps, cellular processes and disease categories using MetaCore™. This allowed us to analyze functionally related genes (for example, genes belonging to a specific biochemical process) as a whole. Table 3 summarizes the significantly changed biological processes for the LC and HC groups. Four key ('master') process categories were affected by cholesterol feeding: lipid metabolism; carbohydrate and amino acid metabolism; transport; immune and inflammatory responses.

**Table 3:** Analysis of processes that are changed significantly upon treatment with dietary cholesterol

			Differentially expressed (%)	
			
Master process	Subprocess (child terms)	Number of genes measured	LC	HC
Lipid metabolism		264	8.7*	24.2*
	Fatty acid metabolism, fatty acid beta-oxidation	8	0.0	50.0*
	Triacylglycerol metabolism	7	0.0	57.1*
	Cholesterol metabolism	27	33.3*	33.3*
	Cholesterol biosynthesis	7	71.4*	57.1*
	Lipoprotein metabolism	18	16.7*	44.4*
	Lipid biosynthesis	105	11.4*	23.8*
				
Immune response		297	3.0	12.1*
	Antigen presentation, exogenous antigen	10	10.0	70.0*
	Antigen processing	17	5.9	35.3*
	Acute-phase response	11	9.1	36.4*
				
General metabolism		3,600	3.3	13.1*
	Cellular polysaccharide metabolism	19	5.3	26.3*
	Polysaccharide biosynthesis	9	0.0	33.3*
	Cofactor metabolism	116	5.2	21.6*
	Regulation of translational initiation	9	0.0	44.4*
	Amino acid metabolism	103	2.9	20.4*
				
Transport		1,119	2.9	14.3*
	Intracellular protein transport	161	3.7	19.9*
	Golgi vesicle transport	16	6.3	37.5*
	Mitochondrial transport	11	18.2*	54.5*

Master processes and their subprocesses (child terms) are listed together with the number of genes measured (third column). Percentages reflect the fraction of genes differentially expressed (within a specific process or pathway) in the LC and HC groups compared to the Con group. Relevant biological processes were identified in GenMAPP by comparison of the set of differentially expressed genes (ANOVA; *P *< 0.01 and FDR < 0.05) with all genes present on the array. *Biological processes with a Z-score >2 and a PermuteP < 0.05.

In the LC group, most significant effects occurred within the master process of lipid metabolism. Important subprocesses (that is, processes in which more than 10% of process-related genes changed significantly) were lipid biosynthesis, lipoprotein metabolism, cholesterol metabolism and cholesterol biosynthesis (Table 3). The overall functional effect of LC treatment can be summarized as a substantial down-regulation of cholesterol and lipid metabolism. This adaptive response of the liver indicates metabolic liver resilience up to doses of 0.25% (w/w) cholesterol.

A further increase of dietary cholesterol (1% w/w; HC group) intensified the changes in gene expression seen with LC treatment, indicating further metabolic adaptation. For example, all individual genes of the cholesterol biosynthesis pathway were down-regulated to a greater extent by HC than by LC treatment (see pathway map in Additional data file 4a): the gene of the rate-limiting enzyme of this pathway, *HMG CoA reductase *(*HDMH*), was down-regulated 2.8-fold and 10.6-fold by LC and HC treatments, respectively. Similarly, genes relevant for lipid and lipoprotein metabolism, *LDL receptor *(LC group, 1.3-fold down-regulated; HC group, 1.9-fold down-regulated) and *lipoprotein lipase *(LC group, 1.8-fold up-regulated; HC group, 5.5-fold up-regulated), were dose-dependently modulated.

Besides marked effects on 'lipid metabolism', HC treatment induced significant changes in the master processes: 'general metabolism', 'transport' and 'immune and inflammatory response' (Table 3). In particular, HC treatment enhanced the subprocesses involved in translational initiation, Golgi vesicle transport, mitochondrial transport, antigen presentation, antigen processing and acute phase response by affecting the expression of more than 35% of the genes in these subprocesses.

**Figure 3:**
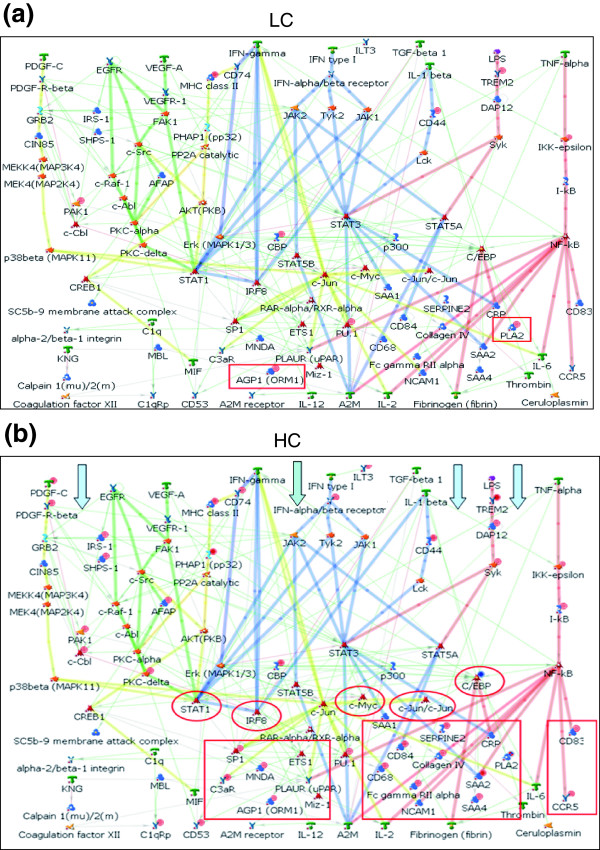
Analysis of the inflammatory pathways activated by the LC and HC diets. A master inflammatory network was generated in MetaCore™ by combining relevant inflammatory pathways. Differentially expressed genes in response to **(a) **LC and **(b) **HC treatment were mapped into this master network. The activation of the network by LC treatment was minimal, whereas HC treatment resulted in a profound activation of specific proinflammatory pathways (marked with blue arrows). Red circles indicate transcriptional node points and red rectangles highlight representative downstream target genes that were up-regulated.

**Figure 4:**
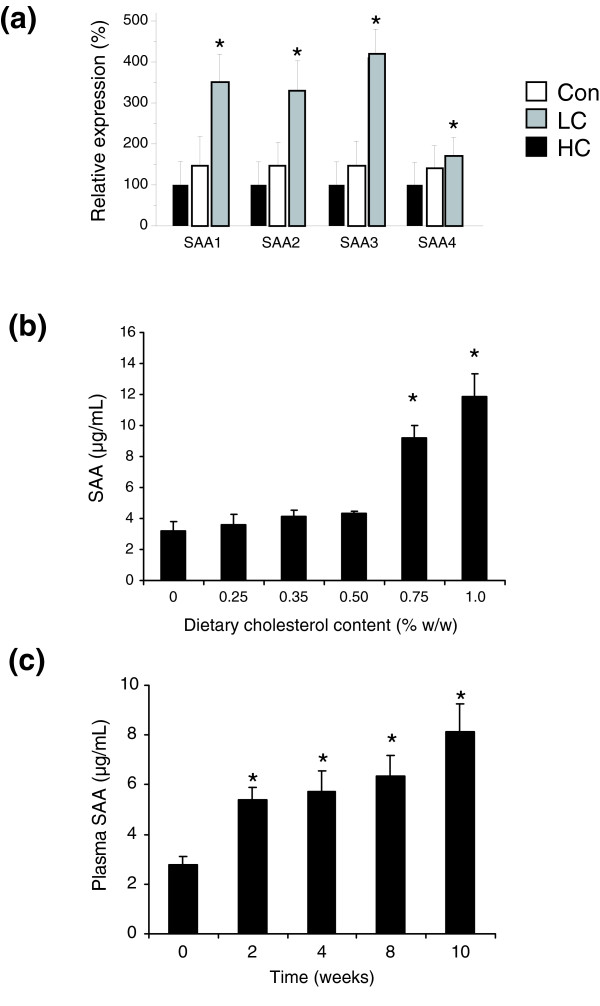
Expression of *SAA *genes. **(a) **All four isotype genes were dose-dependently increased with increasing dietary cholesterol exposure. *Significant compared to Con, *P *< 0.01. **(b) **Plasma SAA levels in response to increasing doses of dietary cholesterol. Female E3L animals (n ≥ 7/condition) were fed the Con diet supplemented with increasing concentrations of cholesterol for 10 weeks. *P < 0.05 versus 0% w/w cholesterol control group. **(c) **Plasma SAA levels in the HC diet (1% w/w cholesterol) fed female E3L animals (*n *= 8) over time. *P < 0.05 versus t = 0.

Significantly, HC but not LC dietary stress activated specific inflammatory pathways (that is, the platelet-derived growth factor (PDGF), interferon-γ (IFNγ), interleukin-1 (IL-1) and tumor necrosis factor-α (TNFα) signaling pathways; Figure 3). Activation of these inflammatory pathways with HC treatment leads to a significant up-regulation of MAP kinases, complement factors and acute phase proteins such as SAA. HC treatment significantly up-regulates, for example, all four *SAA *isotype genes (Figure 4a), which is consistent with the observed changes in SAA protein concentrations in plasma (compare Table 1).

More generally, HC treatment induced many genes, the gene products of which reportedly or putatively initiate or mediate inflammatory events (Additional data file 3), including genes encoding proteases, complement components, chemokines and their receptors, heat shock proteins, adhesion molecules and integrins, acute phase proteins, and inflammatory transcription factors, altogether indicating a profound reprogramming of the liver towards an inflammatory state not observed with LC treatment.

In separate experiments using female E3L mice, the hepatic inflammatory response to cholesterol-feeding was analyzed in more detail, including dose-dependency and time course. Plasma SAA served as a marker and readout of liver inflammation. Feeding of cholesterol at doses up to 0.50% w/w did not alter plasma SAA levels (Figure 4b). At higher cholesterol doses (≥0.75% w/w), plasma SAA levels increased markedly. A time course study with 1% w/w cholesterol (HC diet) showed that plasma SAA levels started to increase after 2 weeks and that plasma SAA levels continued to increase over time (Figure 4c). Together, these refined analyses indicate that the liver is resilient up to a cholesterol dose of about 0.50% w/w (adaptive response) and that the inflammatory response evoked with higher cholesterol concentrations starts within two weeks of starting the HC diet.

Enrichment analysis with disease categories confirmed the activation of many signaling and effector pathways relevant for inflammation and immunity by HC, but not by LC, treatment. The most affected (that is, activated at the gene expression level) disease categories with HC treatment were interrelated cardiovascular disorders and (auto) immune diseases, including cerebral and intracranial arterial diseases, cerebral amyloid angiopathy, hepatocellular carcinoma, and hepatitis (Additional data file 4b).

Altogether, this global analysis shows that the liver responds to a low load of dietary cholesterol mainly by adapting its metabolic program, whereas at a high cholesterol load the liver is much more extensively reprogrammed, and, in addition to metabolic adaptations, expresses genes involved in inflammatory stress.

### Analysis of diet-dependent metabolic changes in liver and plasma

To verify whether the switch from metabolic adaptation (with LC treatment) to hepatic inflammatory stress (with HC treatment) is also reflected at the metabolite level, we performed a comprehensive HPLC/MS-based lipidome analysis (measurement in total of about 300 identified di- and triglycerides, phosphatidylcholines, lysophosphatidylcholines, cholesterol esters) on liver homogenates of Con, LC and HC groups, and corresponding plasma samples.

**Figure 5:**
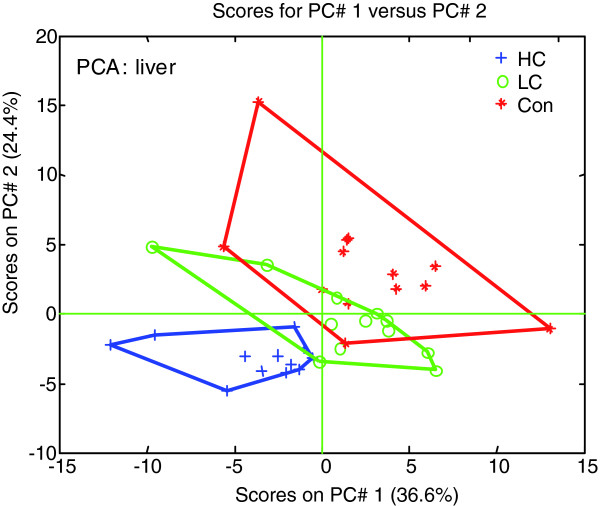
Lipidom analysis of liver homogenates (*n *= 10 per group). Score plot was derived from PCA. The two component model explained 36.6% (principle component 1; PC# 1) and 24.4% (PC# 2) of the variation in the data.

The individual metabolite fingerprints within a treatment group were similar and formed clusters as assessed by principal component analysis (PCA; Figure 5). The clusters of the Con and LC groups overlapped partly, demonstrating that the Con and LC groups have a similar intrahepatic lipid pattern. This indicates that the metabolic adjustments on the gene level in the LC group were efficacious and enabled the liver to cope with moderate dietary stress. The HC cluster did not overlap with the clusters of the Con group, demonstrating that the switch to a proinflammatory liver gene expression profile is accompanied by development of a new metabolic hepatic state, which differs significantly from the metabolic state at baseline (Con group).

### Identification of transcriptional regulators that control the hepatic response to cholesterol

To identify the transcription factors and underlying regulatory mechanisms that govern the hepatic response to LC and HC stress, we performed a combined analysis of the liver transcriptome and metabolome dataset. Functional networks allowed the identification of transcriptional key ('master') regulators relevant for liver resilience and liver inflammation.

The adaptation of hepatic lipid metabolism to LC stress was mainly controlled by retinoid X receptor (RXR), SP-1, peroxisome proliferator activated receptor-α (PPARα), sterol regulatory element binding protein (SREBP)1 and SREBP2 (networks not shown), which are established positive regulators of genes involved in cholesterol biosynthesis [16]. Combined analysis of genome and metabolite datasets revealed that the intrahepatic level of eicosapentaenoic acid, a suppressor of SREBP1 [17], was increased, providing a molecular explanation for the observed down-regulation of genes involved in cholesterol biosynthesis (Additional data file 5).

**Figure 6:**
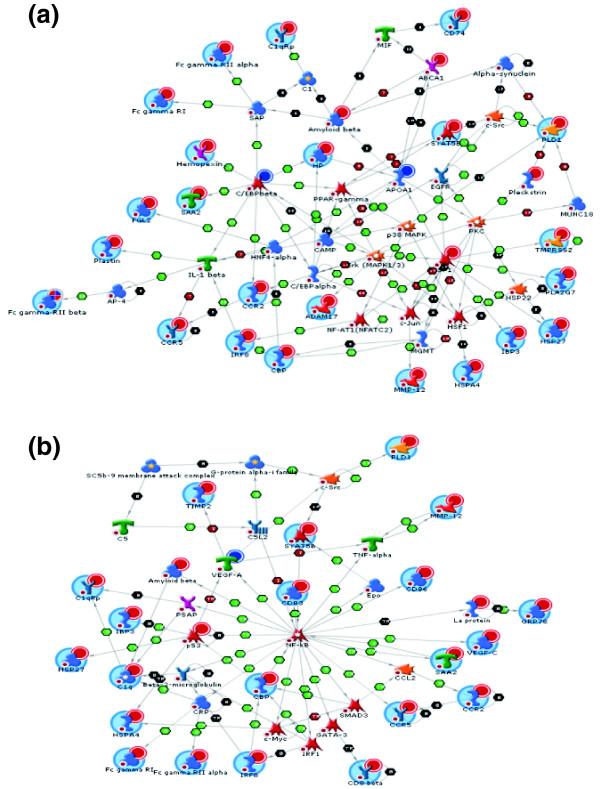
Representative biological network based on differentially expressed genes of the HC group using MetaCore™ network software and the Analyze Network algorithm. Two representative networks are shown: **(a) **the C/EBPβ c-jun network and **(b) **the NF-κB network. A legend for the biological networks is provided in Additional data file 7d. Red dots in the right corner of a gene indicate up-regulation and blue dots down-regulation.

A subsequent network analysis of HC-modulated genes allowed the identification of transcription factors that mediate the evolution of hepatic inflammation and are ultimately responsible for the effects on the process level. HC-evoked changes require specific transcriptional master regulators, some of which are established in this context (nuclear factor kappa B (NF-κB), activator protein-1 (AP-1), CAAT/enhancer-binding protein (C/EBP)β, p53), and others that are new (CREB-binding protein (CBP), hepatocyte nuclear factor-4α(HNF4α), SP-1, signal transducer and activator of transcription-3/-5 (STAT-3/-5), Yin Yang-1 (YY1); Figure 6 and Additional data file 6).

Consistent with this, the identified transcription factors control the expression of genes encoding acute phase response proteins, complement factors, growth factors, proteases, chemokine receptors and factors stimulating cell adhesion, as confirmed by data mining. Most importantly, HC treatment induced genes whose products can act extracellularly (Additional data file 7) and possess reportedly pro-atherogenic properties. Examples include complement components (C1qb, C1qR, C3aR1, C9), chemoattractant factors (ccl6, ccl12, ccl19), chemoattractant receptors (CCR2, CCR5), cytokines inducing impaired endothelial barrier function (IFN-γ), adhesion regulators (integrin β2, integrin β5, CD164 antigen/sialomucin, junction adhesion molecule-2), growth factors (PDGF, vascular endothelial growth factor (VEGF)-C, transforming growth factor (TGF)-β), proteases involved in matrix remodeling during atherogenesis (cathepsins B, L, S and Z; matrix metalloprotease-12), and cardiovascular risk factors/inflammation markers (haptoglobin, orosomucoid 2, fibrinogen-like protein 2, α1-microglobulin). This upregulation of pro-atherogenic candidate genes in the HC group is consistent with the observed enhanced early atherosclerosis found in this group.

Expansion of the lipid and inflammatory networks revealed that hepatic lipid metabolism is linked to the hepatic inflammatory response via specific transcriptional regulators that control both processes. Among these dual regulators were CBP, C/EBPs, PPARα and SP-1 (Table 4). The presence of molecular links between lipid metabolism and inflammation raises the possibility that specific intervention with an anti-inflammatory compound may, in turn, affect plasma cholesterol levels. In a first attempt to test this possibility, female E3L mice were fed a HC diet to increase plasma cholesterol levels (from 5.3 mM to 19.3 mM) and systemic inflammation (SAA from 1.7 μg/ml to 9.2 μg/ml). Then, animals were treated with the same HC diet supplemented with salicylate, an inhibitor of NF-κB signaling, or vehicle. While plasma cholesterol and SAA levels remained elevated in the vehicle-treated group, the salicylate-treated group displayed significantly lower plasma SAA levels (7.7 μg/ml; *P *< 0.05) and significantly reduced cholesterol levels (9.9 mM; *P *< 0.05) demonstrating that specific intervention into the inflammatory component does indeed affect plasma cholesterol.

**Table 4:** Identified master regulators that control inflammatory reprogramming of the liver

Transcription factor	Regulator of/node point for	Example of downstream effects
AP-1 (c-jun/c-fos)	Inflammation	Mmp-12, col1a1, hsp27
CREP binding protein (CBP)	Lipid, inflammation, immune response, cell proliferation	Very broadly acting coactivator (can bind to SREBPs)
C/EBPs	Lipid, inflammation, energy metabolism	Acute phase genes (for SAA, CRP, fibrinogen), hepatic gluconeogenesis and lipid homeostasis, energy metabolism (PEPCK, FAS), TGF-β signaling
Forkhead transcription factor FOXO1	Lipid, inflammation/proliferation	Glycolysis, pentose phosphate shunt, and lipogenic and sterol synthetic pathways, LPL (via SHP)
NF-κB	Inflammation	SAA, CD83, CD86, CCR5, VEGF-C
PPARα/RXRα	Lipid, inflammation	LPL, ABCA1, macrophage activation, glucose homeostasis
p53	Inflammation	HSP27, HSPA4, IFI16, IBP3, RBBP4
SMAD3	Inflammation	Proteases and growth factors (via TGF-β signaling)
SP-1	Lipid, inflammation	ABCA1, ICAM-1, cellular matrix genes COL1A1, COL1A2
SREBP-1/-2	Lipid, inflammation	Sterol biosynthesis genes, LDLR, link to C/EBPα
STAT1/3/5	Inflammation	Acute phase genes
YY1	Inflammation/proliferation	Inflammatory response genes (SAA, vWF, CCR5), cellular matrix genes

Biological networks were generated using MetaCore™ software and transcriptional master regulators were identified in significant gene networks (*P *< 0.05).

## Discussion

Development of atherosclerotic lesions requires a lipid component (hypercholesterolemia) and an inflammatory component. In the present study, we demonstrate that high dose dietary cholesterol (HC diet) strongly induces early atherosclerotic lesion formation in a humanized model for atherosclerosis, E3L mice. This is not, or is only slightly, the case with a cholesterol-free (Con) or low dose cholesterol (LC) diet. Importantly, Con, LC, and HC diets dose-dependently increase plasma cholesterol levels, but only HC treatment induces a marked systemic inflammatory response, which precedes lesion formation and is related to liver inflammation. We employed newly developed (functional) systems biology technologies to unravel how increasing the dose of dietary cholesterol affects liver homeostasis and evokes hepatic inflammation. The following important findings were made. The liver absorbs escalating doses of dietary cholesterol primarily by adjusting the expression level of genes involved in lipid metabolism, as revealed by advanced gene expression analysis. This metabolic resilience is confirmed by analysis of metabolites in liver. At high doses of dietary cholesterol, the liver also develops an inflammatory stress response, which is characterized by up-regulation of pro-atherogenic candidate genes and activation of (at least four distinct) inflammatory pathways. The evolution of hepatic inflammation involves specific transcriptional regulators, several of which have been newly identified in this study. Interestingly, some of these transcription factors have a dual role and control both hepatic lipid metabolism and hepatic inflammation, indicating that the same regulatory mechanisms underlie these processes and thereby link the two processes.

The present study delineates, to our knowledge for the first time, the genome-wide response of the liver to increasing doses of dietary cholesterol, with specific attention to inflammatory processes, and in relation to early atherosclerotic lesion formation. The liver responds to moderate elevations in dietary cholesterol (LC diet) by adjusting major metabolic processes related to lipid metabolism. For example, the expression of genes involved in endogenous cholesterol synthesis (for example, *HMG-CoA reductase*) and cholesterol uptake from plasma (for example, *LDLR*) is diminished. At high loads of dietary cholesterol (HC diet), the liver strives for homeostasis by intensifying the changes in gene expression observed with the LC diet. Similar dose-dependent effects of dietary cholesterol have been reported by others [18] but the number of studies that assess dose-dependent effects of dietary cholesterol is relatively small and analyses are restricted to a limited number of genes. Our genome-wide approach is comprehensive and demonstrates that metabolic processes as a whole are adjusted at the level of gene expression.

Importantly, the adjustment at the gene level is efficacious only up to a certain degree of cholesterol stress: while low loads of cholesterol are fully absorbed (consider the comparable intrahepatic lipidome fingerprints for the LC and Con groups), exposure to high loads of dietary cholesterol in the HC group significantly altered the liver lipidome, despite further intensified adjustment of gene expression. Our combined analysis of genes and functional readouts (lipid metabolites) clearly demonstrates that a dose of 1% w/w of cholesterol, which is typically used to induce experimental dyslipidemia and atherosclerosis in mice [13,19,20], is an extreme condition because the metabolic resilience of the liver is already overstretched.

Concomitant with the adjustment of metabolic genes to HC dietary stress, HC treatment also evokes a hepatic inflammatory response. The development of an inflammatory gene expression profile upon feeding of a diet containing cholesterol has also been reported by others. For example, Tous *et al*. [21] showed that atherosclerosis-prone apoE-/- mice receiving a high fat/high cholesterol diet develop an impairment of liver histology consisting of fat accumulation, macrophage proliferation, and inflammation, and that there is a chronological and quantitative relationship between liver impairment and the formation of atheromatous lesions. Vergnes *et al*. [22] showed that cholesterol and cholate components of the atherogenic diet have distinct pro-atherogenic effects on gene expression and particularly that cholesterol is required for induction of genes involved in acute inflammation in C57BL/6J mice. Recinos *et al*. [23] reported that liver gene expression in LDLR-/- mice is associated with diet and lesion development and demonstrated the induction of components of the alternative component pathway. Zabalawi *et al*. [24] showed the induction of fatal inflammation in LDLR-/- and ApoAI-/-LDLR-/- mice fed dietary fat and cholesterol. However, the exact molecular inflammatory pathways switched on/off by dietary cholesterol have remained unknown. While some of the above studies employing microarray analysis have examined some of the individual components of the inflammatory response to cholesterol, we have set out to generate a holistic profile of the complex, interrelated nature of the response of the liver to cholesterol. Advanced pathway analysis combined with functional network building enabled us to unravel four key inflammatory pathways (IFNγ, TNFα, IL-1, and PDGF pathways) that play central roles in the evolution of cholesterol-induced inflammation in the liver. Further research is necessary to resolve the sequence of events over time (for example, which pathway is switched on first). Remarkably, these pathways are also critical for lesion development in the vessel wall, suggesting that the inflammatory response to cholesterol stress described herein for the liver may involve similar routes in other tissues and, as such, has more general significance.

Our results suggest that hepatic inflammatory response may be causatively related to lesion initiation in the aorta, because pro-atherogenic candidate genes (that is, genes encoding candidate inflammatory components reportedly or putatively involved in early lesion formation) were found to be upregulated specifically in the HC group but not, or only slightly, in the LC group. The presence of a 'hepatic source' for inflammatory factors in HC stress may also explain the observed exponential (rather than linear) increase in lesion formation seen with increasing dietary cholesterol loads [11,12]. Consistent with this notion is the view that the inflammatory arm of atherogenesis is a principle driving force of lesion development.

An inflammatory reprogramming of the liver has also been observed in C57BL/6J mice treated with a 1.25% w/w cholesterol diet resulting in total plasma cholesterol concentrations of 3.6 mM [22], that is, a level comparable to the Con group in our study. Unlike that in E3L mice, the total plasma cholesterol in C57BL/6J mice is mainly confined to HDL, an anti-atherogenic, anti-inflammatory lipoprotein facilitating transport of cholesterol from the periphery back to the liver. The fact that mice with a strongly different lipoprotein profile (E3L, LDLR-/- and C57BL/6) show a similar hepatic inflammatory response to cholesterol feeding indicates that the observed inflammatory effect of dietary cholesterol is a general phenomenon and not restricted to the model of dysbetalipoproteinemia used herein. Also, it suggests that the influx of dietary cholesterol into the liver (via chylomicrons) rather than plasma cholesterol is key to the inflammatory response of the liver. This supposition would also be in accord with the rapidity of the effect: in a time-resolved analysis of plasma SAA during atherogenesis, we report here a strong elevation of plasma SAA within two weeks of cholesterol-feeding in female E3L mice. This is also in line with the inflammatory reprogramming of C57BL6/J mice within three weeks [22] and clearly demonstrates that the hepatic inflammatory response precedes the formation of atherosclerotic lesions, suggesting that dietary cholesterol can be an important trigger and a possible source of the inflammatory component of atherosclerotic disease. In the present study, the liver function markers ALAT and ASAT remained within the normal levels stipulated for the function criteria for donor livers [25], indicating normal liver function under the experimental conditions applied in this study. Our results do not exclude the possibility, however, that sterols may oxidize and become toxic and that the oxidized sterols contribute, at least partly, to the inflammatory effects observed by us and others.

Inflammation may also arise from established risk factors other than high plasma cholesterol (for example, hypertension, diabetes/hyperglycemia). Dietary glucose can modulate the mRNA expression and serum concentrations of immune parameters but these alterations rapidly normalize in normoglycemic subjects [26]. In the case of an impaired metabolic state, however, postprandial hyperglycemia increases the magnitude and duration of systemic inflammatory responses, which probably promotes the development of cardiovascular disease.

Our results show that the evolution of hepatic inflammation is controlled by specific transcriptional regulators, some of which are well known in the context of cholesterol-inducible inflammation (SREBPs, NF-κB, AP-1, C/EBPs), while others have been newly identified in the present study (CBP, HNF4α, SP-1, STAT-3/-5, YY1). Interestingly, some of these factors may also represent molecular links between lipid/cholesterol metabolism and inflammation. Supportive evidence for an interrelationship between liver metabolism and inflammation also comes from pharmacological intervention studies. On the one hand, cholesterol-lowering drugs reduce the general inflammatory status and the expression of liver-derived inflammation markers in E3L mice (compare to the pleiotropic effects of statins) [7,14,27] The anti-inflammatory IKKβ-inhibiting compound salicylate [28,29] reduces plasma cholesterol in the same mouse model (this paper) indicating that modulation of cholesterol levels via inflammation may be possible as well. A hypocholesterolemic effect of salicylate has also been reported in catfish [30] and salicylate was found to inhibit hepatic lipogenesis in isolated rat hepatocytes *in vitro *[31]. Prigge and Gebhard [32] showed that acetylsalicylate (aspirin), a classical inhibitor of COX1 and COX2 [29], induces biliary cholesterol secretion in the rat, an effect that may contribute to the cholesterol-lowering effect seen with compounds of the salicylate category: in diabetic human subjects, very high doses of aspirin (around 7 g/d) were associated with a 15% reduction of total plasma cholesterol and CRP [29].

## Conclusion

We demonstrate that dietary cholesterol is not only a lipid risk factor but also a trigger of hepatic inflammation and, as such, also involved in the evolution of the inflammatory arm of atherosclerotic disease. A certain degree of genetic resilience and elasticity allows the liver to cope with moderate cholesterol stress, but high loads of cholesterol result in an inflammatory pro-atherogenic response (involving specific pathways and transcriptional regulators), which enhances early lesion formation. Our findings that cholesterol and inflammation are closely linked via specific transcriptional master regulators might lead to new strategies for future therapeutic intervention.

## Materials and methods

### Animals and diets

Female E3L mice were used at the age of 12 weeks for all experiments. Animal experiments were approved by the Institutional Animal Care and Use Committee of The Netherlands Organization for Applied Scientific Research (TNO) and were in compliance with European Community specifications regarding the use of laboratory animals.

A group of E3L mice (*n *= 17) was treated with a cholesterol-free diet (diet T; Hope Farms, Woerden, The Netherlands) for 10 weeks (Con group). The major ingredients of diet T (all w/w) were cacao butter (15%), corn oil (1%), sucrose (40.5%), casein (20%), corn starch (10%) and cellulose (6%). Two other groups (*n *= 17 each) received the same diet but supplemented with either 0.25% w/w cholesterol (LC group) or 1.0% w/w cholesterol (HC group). After ten weeks of diet feeding, animals were euthanized under anesthesia to collect livers, hearts and aortas. Tissues were snap-frozen in liquid nitrogen and stored at -80°C until use.

To assess the effect of salicylate on plasma levels of inflammation markers and cholesterol, two groups of female E3L mice (*n *= 10; 12 weeks old) were treated with the HC diet for 3 weeks. Then, HC dietary treatment was either continued (vehicle control group) or animals were fed HC supplemented with 0.12% w/w salicylate (equaling a dose of 145 mg/kg/day) for 8 weeks. Plasma samples were obtained by tail bleeding without fixation of the test animals to minimize stress.

### Analyses of plasma lipids and proteins

Total plasma cholesterol and triglyceride levels were measured after 4 h of fasting, using kits No.1489437 (Roche Diagnostics, Almere, The Netherlands) and No.337-B (Sigma, Aldrich Chemie BV, Zwijndrecht, The Netherlands) [33]. For lipoprotein profiles, pooled plasma was fractionated using an ẢKTA FPLC system (Pharmacia, Roosendaal, The Netherlands) [9]. The plasma levels of SAA were determined by ELISA as reported [14]. Plasma ALAT and ASAT levels were determined spectrophotometrically using a Reflotron system (Roche Diagnostics) [9].

For lipiodomics analysis of liver homogenates and plasma samples, electrospray liquid chromatography mass spectroscopy (LC-MS) analysis was applied [34]. Briefly, samples (5 μl) were incubated with 200 μl isopropanol and a mixture of internal standards (heptadecanoyl-lysophosphatidylcholine, di-lauroyl-phosphatidylcholine, heptadecanoyl-cholesterol and tri-heptadecanoyl-glycerol; Sigma, St Louis, MO, USA)). After vortexing, the lipids were extracted and isolated by centrifugation (lipids in isopropanol phase). Electrospray LC-MS lipid analysis was performed on a Thermo LTQ apparatus equipped with a Surveyor HPLC system (Thermo Electron, San Jose, CA, USA). The samples were measured in fully randomized sequences. Quality control samples, prepared from a single pool of E3L mouse reference tissue, were analyzed at regular intervals (bracketing 10 samples). The LC-MS raw data files were processed using a software developed by TNO (IMPRESS) to generate comprehensive peak tables (m/z value, retention time and peak area). Data were then subjected to retention time alignment of peaks, internal standard correction of peak areas and quality control resulting in a final lipidomics dataset.

The obtained lipidomics dataset was analyzed and visualized by PCA essentially as described [35]. Prior to analysis, the data were mean-centered and auto-scaled to ensure an equal contribution of all lipid measurements to the PCA-model.

### Analyses of atherosclerosis

Hearts were fixed and embedded in paraffin to prepare serial cross sections (5 μm thick) throughout the entire aortic valve area for (immuno) histological analysis. Cross sections were stained with hematoxylin-phloxine-saffron, and atherosclerosis was analyzed blindly in four cross-sections of each specimen (at intervals of 30 μm) as reported [14,36]. QWin-software (Leica) was used for morphometric computer-assisted analysis of lesion number, lesion area, and lesion severity as described in detail elsewhere [7]. Significance of difference was calculated by one-way analysis of variance (ANOVA) test followed by a least significant difference *post hoc *analysis using SPSS 11.5 for Windows (SPSS, Chicago, IL, USA). The level of statistical significance was set at α < 0.05.

### Nucleic acid extraction and gene expression analysis

Total RNA was extracted from individual livers (*n *= 5 per group) using RNAzol (Campro Scientific, Veenendaal, The Netherlands) and glass beads according to the manufacturer's instructions. The integrity of each RNA sample obtained was examined by Agilent Lab-on-a-chip technology using the RNA 6000 Nano LabChip kit and a bioanalyzer 2100 (both Agilent Technologies, Amstelveen, The Netherlands). The quality control procedure is described in Additional data file 8. The One-Cycle Target Labeling and Control Reagent kit (Affymetrix #900493) and the protocols optimized by Affymetrix were used to prepare biotinylated cRNA (from 5 μg of total RNA) for microarray hybridization (*n *= 5 per group). The quality of intermediate products (that is, biotin-labeled cRNA and fragmented cRNA) was again controlled using the RNA 6000 Nano Lab-on-a-chip and bioanalyzer 2100. Microarray analysis was carried out using an Affymetrix technology platform and Affymetrix GeneChip^® ^mouse genome 430 2.0 arrays (45,037 probe sets; 34,000 well-characterized mouse genes). Briefly, fragmented cRNA was mixed with spiked controls, applied to Affymetrix Test chips, and good quality samples were then used to hybridize with murine GeneChip^® ^430 2.0 arrays. The hybridization, probe array washing and staining, and washing procedures were executed as described in the Affymetrix protocols, and probe arrays were scanned with a Hewlett-Packard Gene Array Scanner (Leiden Genome Technology Center, Leiden, The Netherlands).

### Gene expression data analysis

Raw signal intensities were normalized using the GCRMA algorithm (Affylm package in R). Datasets are freely accessible online through ArrayExpress [37]. Normalized signal intensities below 10 were replaced by 10. Probe sets with an absent call in all arrays were removed before further analysis of the data. RT-PCR was performed essentially as described [27,38] to validate and confirm differences in gene expression between the treatment groups.

Statistical analysis was performed in BRB ArrayTools (Dr Richard Simon and Amy Peng Lam [39]). Con, LC and HC groups were tested for differentially expressed genes using class comparisons with multiple testing corrections by estimation of false discovery rate (FDR). Differentially expressed genes were identified at a threshold for significance of α < 0.01 and a FDR < 5%. Within the set of differentially expressed genes, a Student's *t*-test was carried out to analyze differential expression of individual genes between the cholesterol-fed groups and the Con group. Differences of *P *< 0.01 versus Con were considered significant.

For biological interpretation of the differentially expressed genes, software tools GenMAPP and MetaCore™ (GeneGo Inc., St Joseph, MI, USA) were used. Enrichment of biological processes (GO annotation) was analyzed in GenMAPP, biological processes in GenMAPP with a Z-score >2 and PermuteP < 0.05 were considered as significantly changed. In MetaCore™, enrichment analysis [40] of four independent ontologies was performed. In addition to biological process gene ontology, data were also analyzed in canonical pathway maps, GeneGo-cellular processes and disease categories.

Distribution by canonical pathway maps reveals the most significant signaling and/or metabolic pathways. Experimental data are visualized as red/blue thermometers pointing up/down, and signifying up/down-regulation of the map objects. Distribution by GeneGo processes provides the most significant functional process categories enriched with experimental data. GeneGo processes represent comprehensive pre-built process-specific networks, in which all objects are interconnected by experimentally validated interactions. The up- and down-regulated genes are visualized as red or blue circles, respectively. The disease categories represent sets of genes associated with certain diseases. Gene enrichment analysis shows the relative enrichment of the up- and down-regulated genes with the genes from different disease categories. As in the case of process enrichment, this procedure is carried out by p value distribution.

The biological networks were assembled from manually curated protein-protein, protein-DNA and protein-ligand (metabolite) interactions, which are accumulated in the MetaCore™ database. Each edge or link on the network is based on small-experiment data referenced in the corresponding literature. The legend for MetaCore™ Networks from the MetaCore™ guideline is provided in Additional data file 6d. For the generation of functional networks, transcriptome and metabolome datasets were merged, allowing combined analysis. Networks were generated using the Shortest path (SP) algorithm, which links the nodes from experimental datasets by the shortest directed graphs, allowing up to two additional steps using interactions and nodes from the MetaCore™ database. To present most of the relevant network data on the same figure, we used the add/expand function and the Merge Networks feature. The resulting networks provide links based on the known interaction data not only between the nodes from the query data set(s), but also between the nodes that regulate the given genes or metabolites. Network nodes with available experimental data are distinguished with red or blue circles, representing up- or down-regulation, respectively.

## Abbreviations

ALAT, alanine aminotransferase; AP, activator protein; ASAT, aspartate aminotransferase; CBP, CREB-binding protein; C/EBP, CAAT/enhancer-binding protein; Con, cholesterol-free; CRP, C-reactive protein; E3L, ApoE*3Leiden transgenic; FDR, false discovery rate; GO, Gene Ontology; HC, high-cholesterol; HDL, high-density lipoprotein; HNF, hepatocyte nuclear factor; IFN, interferon; IL, interleukin; LC, low-cholesterol; LC-MS, liquid chromatography mass spectroscopy; LDL, low-density lipoprotein; NF-κB, nuclear factor kappa B; PCA, principal component analysis; PDGF, platelet-derived growth factor; PPAR, peroxisome proliferator activated receptor; RXR, retinoid X receptor; SAA, serum amyloid A; SREBP, sterol regulatory element binding protein; STAT, signal transducer and activator of transcription; TGF, transforming growth factor; TNF, tumor necrosis factor; VLDL, very low-density lipoprotein; YY, Yin Yang.

## Authors' contributions

RK provided the conceptual background to the analysis, interpreted the results and wrote the manuscript. LV did the *in vivo *atherosclerosis studies, performed the assays and interpreted the data. MvE performed the computational analysis including biological processes and assisted with manuscript writing. YN coordinated the software development and the multidimensional analysis of biological processes using (pathway) networks. NC supervised the work and coordinated the lipidomics and genomics analyses. EV developed the lipidomics methodology and performed the lipidomics measurements. AS coordinated the multivariate statistical analysis and drafted the manuscript. HH helped with data interpretation and evaluated the manuscript. SZ performed animal experiments and quantified plasma inflammation markers. GS participated in designing the experiment and manuscript writing. VK developed the tools for multidimensional data analyses and performed computations. TN assisted with the preparation of the figures of networks and pathways. AM assisted in data interpretation and bioinformatical techniques for gene ontology analyses. EH participated in designing the study and manuscript preparation. JG coordinated the development of the metabolomics technologies and critically evaluated the manuscript. BO led the bioinformatical analyses, developed the concepts for integrated data analysis and drafted the manuscript. TK initiated the study, interpreted the data and helped with manuscript writing. All authors read and approved the final manuscript.

## Additional data files

The following additional data are available with the online version of this paper. Additional data file 1 shows the exponential positive correlation between atherosclerotic lesion area and total plasma cholesterol in female E3L mice. Additional data file 2 shows the validation and confirmation of Affymetrix microarray gene expression data by RT-PCR analysis. Additional data file 3 is a table of the genes (including GenBank identification number and the gene symbol) that are differentially expressed with increasing doses of dietary cholesterol. Additional data file 4 shows the canonical pathway analysis for cholesterol metabolism and analysis of the gene expression data based on GO annotation with disease categories (MetaCore™ software, GeneGO). Additional data file 5 shows the comprehensive network analysis (functional OMICs analysis) by merging gene expression datasets with the metabolite datasets using MetaCore™ network software. Additional data file 6 shows the biological networks of differentially expressed genes in the HC group allowing the identification of transcriptional master regulators. Additional data file 7 is a table lisitng cholesterol-induced factors with reported extracellular function. Additional data file 8 describes the quality control analysis steps for RNA samples prior to hybridization on Affymetrix microarrays usig Agilent Lab-on-a-chip technology.

## Supplementary Material

Additional data file 1 Exponential positive correlation between atherosclerotic lesion area and total plasma cholesterol in female E3L mice. Click here for file

Additional data file 2 Validation and confirmation of Affymetrix microarray gene expression data by RT-PCR analysis. Click here for file

Additional data file 3 Genes (including GenBank identification number and the gene symbol) that are differentially expressed with increasing doses of dietary cholesterol. Click here for file

Additional data file 4 Canonical pathway analysis for cholesterol metabolism and analysis of the gene expression data based on GO annotation with disease categories (MetaCore™ software, GeneGO). Click here for file

Additional data file 5 Comprehensive network analysis (functional OMICs analysis) by merging gene expression datasets with the metabolite datasets using MetaCore™ network software. Click here for file

Additional data file 6 Biological networks of differentially expressed genes in the HC group allowing the identification of transcriptional master regulators. Click here for file

Additional data file 7 Cholesterol-induced factors with reported extracellular function. Click here for file

Additional data file 8 Quality control analysis steps for RNA samples prior to hybridization on Affymetrix microarrays usig Agilent Lab-on-a-chip technology. Click here for file
