# Supplementary material for: Atherosclerosis and liver inflammation induced by increased dietary cholesterol intake: a combined transcriptomics and metabolomics analysis
Source: Genome Biol. 2007 Sep 24;8(9):R200. doi: 10.1186/gb-2007-8-9-r200 (PMC2375038; doi:10.1186/gb-2007-8-9-r200)
Supplement: Additional data file 1 — Exponential positive correlation between atherosclerotic lesion area and total plasma cholesterol in female E3L mice. [file gb-2007-8-9-r200-S1.ppt]

## Slide 1
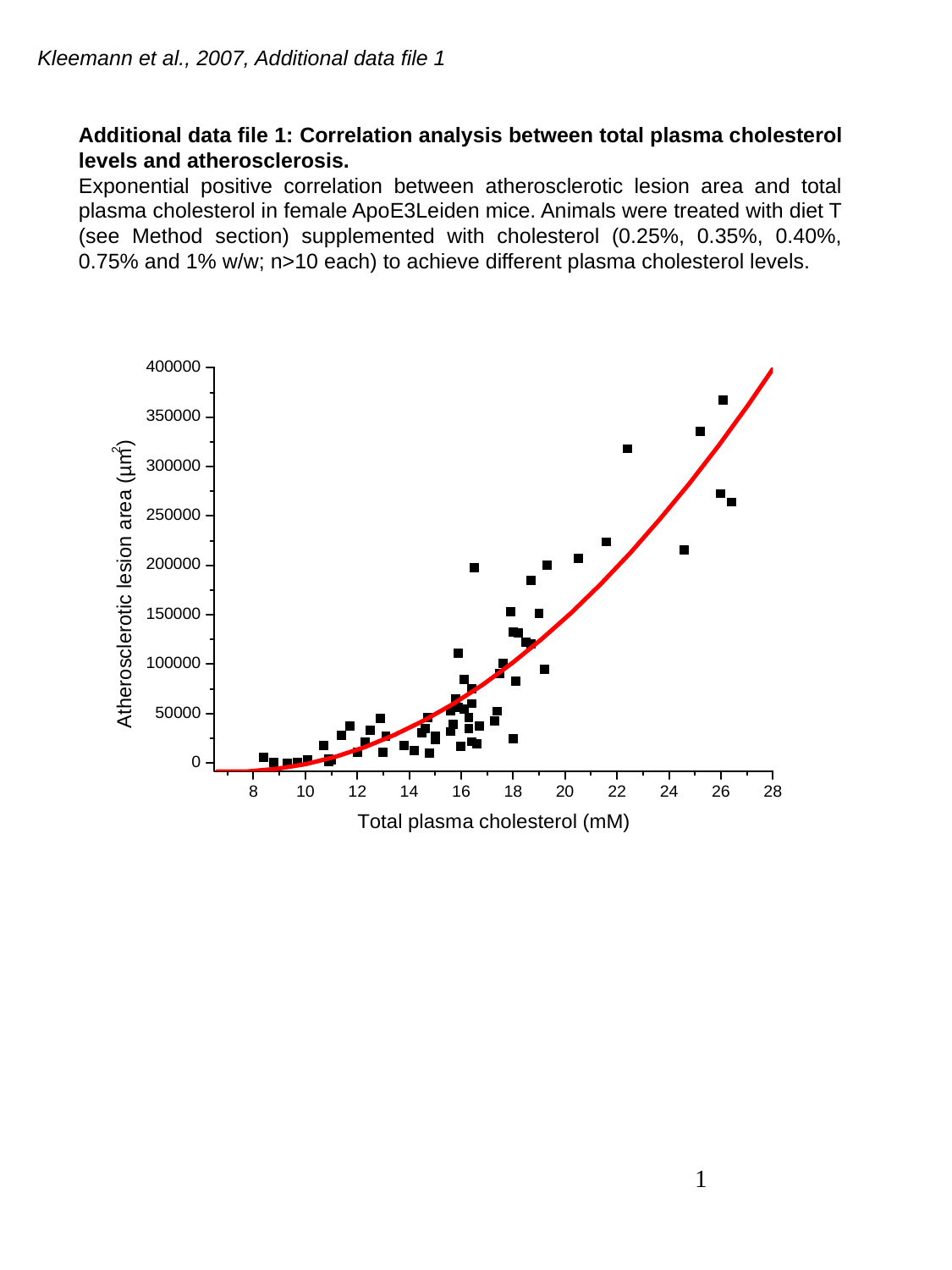

Kleemann et al., 2007, Additional data file 1
Additional data file 1: Correlation analysis between total plasma cholesterol levels and atherosclerosis.
Exponential positive correlation between atherosclerotic lesion area and total plasma cholesterol in female ApoE3Leiden mice. Animals were treated with diet T (see Method section) supplemented with cholesterol (0.25%, 0.35%, 0.40%, 0.75% and 1% w/w; n>10 each) to achieve different plasma cholesterol levels.
1
