# Supplementary material for: Atherosclerosis and liver inflammation induced by increased dietary cholesterol intake: a combined transcriptomics and metabolomics analysis
Source: Genome Biol. 2007 Sep 24;8(9):R200. doi: 10.1186/gb-2007-8-9-r200 (PMC2375038; doi:10.1186/gb-2007-8-9-r200)
Supplement: Additional data file 3 — Genes (including GenBank identification number and the gene symbol) that are differentially expressed with increasing doses of dietary cholesterol. [file gb-2007-8-9-r200-S3.doc]

*Kleemann et al., 2007 Additional data file 3*

**Additional data file 3:** **Dose-dependent effect of dietary cholesterol on expression of hepatic inflammatory genes.**

A selection of inflammatory genes significantly up-regulated (red) or down-regulated (green) by cholesterol feeding. The effect of LC and HC treatment is expressed as a fold change compared to Con. Only genes with significant (P<0.01 vs. Con) differential expression in LC and/or HC are listed together with their GenBank identification number and the gene symbol.

|  | **LC** | | **HC** | |  |  |
| --- | --- | --- | --- | --- | --- | --- |
| **Gene name** | **Fold change** | **P-value** | **Fold change** | **P-value** | **GenBank-id number** | **Gene symbol** |
| orosomucoid 3 | 2.09 | 0.0022 | 4.68 | 0.0005 | NM_013623 | Orm3 |
| serum amyloid A 3 | 1.45 |  | 4.11 | 0.0002 | NM_011315 | Saa3 |
| orosomucoid 2 | 1.76 | 0.0060 | 4.05 | 0.0001 | NM_011016 | Orm2 |
| serum amyloid A 1 | 1.48 |  | 3.55 | 0.0029 | NM_009117 | Saa1 |
| serum amyloid A 1 | 1.48 |  | 3.45 | 0.0013 | NM_011314 | Saa1 |
| serum amyloid A 2 | 1.39 |  | 3.24 | 0.0025 | NM_011314 | Saa2 |
| annexin A2 | 1.35 |  | 3.07 | 0.0000 | NM_007585 | Anxa2 |
| insulin induced gene 2 | 1.06 |  | 3.06 | 0.0001 | AV257512 | Insig2 |
| CD83 antigen | 1.66 |  | 3.03 | 0.0007 | NM_009856 | Cd83 |
| histocompatibility 2, class II antigen E beta | 1.62 |  | 2.93 | 0.0000 | NM_010382 | H2-Eb1 |
| insulin-like growth factor binding protein 5 | 1.51 |  | 2.81 | 0.0001 | BF225802 | Igfbp5 |
| insulin induced gene 2 | 1.24 |  | 2.78 | 0.0000 | AV257512 | Insig2 |
| heat shock protein 1 | 1.25 |  | 2.75 | 0.0023 | U03561 | Hspb1 |
| chemokine (C-C motif) ligand 6 | 1.24 |  | 2.67 | 0.0001 | BC002073 | Ccl6 |
| histocompatibility 2, class II antigen A, beta 1 | 1.39 |  | 2.64 | 0.0001 | M15848 | H2-Ab1 |
| histocompatibility 2, class II antigen A, alpha /// class II antigen E alpha | 1.39 |  | 2.61 | 0.0001 | AF119253 | H2-Aa /// H2-Ea |
| Histocompatibility 2, class II antigen A, alpha, mRNA (cDNA clone MGC:25391 IMAGE:3670758) | 1.37 |  | 2.59 | 0.0001 | AV018723 | H2-Aa |
| interferon regulatory factor 8 | 1.55 |  | 2.58 | 0.0000 | BG069095 | Irf8 |
| histocompatibility 2, class II antigen A, beta 1 | 1.42 |  | 2.54 | 0.0002 | M15848 | H2-Ab1 |
| chemokine (C-C motif) ligand 6 | 1.21 |  | 2.52 | 0.0002 | AV084904 | Ccl6 |
| fibrinogen-like protein 2 | 1.78 | 0.0008 | 2.51 | 0.0003 | BF136544 | Fgl2 |
| complement component 3a receptor 1 | 1.28 |  | 2.48 | 0.0001 | NM_009779 | C3ar1 |
| histocompatibility 2, class II antigen A, beta 1 | 1.38 |  | 2.47 | 0.0002 | NM_010379 | H2-Ab1 |
| histocompatibility 2, class II antigen A, alpha | 1.39 |  | 2.44 | 0.0001 | BE688749 | H2-Aa |
| lymphocyte antigen 86 | 1.23 |  | 2.26 | 0.0000 | NM_010745 | Ly86 |
| heat shock protein 1 | 1.03 |  | 2.24 | 0.0044 | NM_013560 | Hspb1 |
| histocompatibility 2, class II, locus Mb1 /// locus Mb2 | 1.21 |  | 2.19 | 0.0009 | NM_010388 | H2-DMb1 /H2-DMb2 |
| Ia-associated invariant chain | 1.28 |  | 2.11 | 0.0007 | BC003476 | Ii |
| latent transforming growth factor beta binding protein 3 | 1.56 | 0.0028 | 2.05 | 0.0044 | BB324823 | Ltbp3 |
| complement component 3a receptor 1 | 1.39 |  | 2.04 | 0.0002 | BB333624 | C3ar1 |
| junction adhesion molecule 2 | 1.55 |  | 2.03 | 0.0008 | AU016127 | Jam2 |
| endothelial differentiation, sphingolipid G-protein-coupled receptor, 3 | 1.33 | 0.0060 | 1.96 | 0.0003 | AV238324 | Edg3 |
| macrophage activation 2 like | 1.77 | 0.0016 | 1.88 | 0.0004 | BM241485 | Mpa2l |
| Fc receptor, IgG, high affinity I | 1.47 |  | 1.85 | 0.0008 | AF143181 | Fcgr1 |
| interferon gamma inducible protein 47 | 1.30 |  | 1.85 | 0.0000 | NM_008330 | Ifi47 |
| glycoprotein 49 A /// leukocyte immunoglobulin-like receptor, subfamily B, member 4 | 1.29 |  | 1.85 | 0.0035 | U05264 | Gp49a /// Lilrb4 |
| ring finger protein 125 | 1.39 |  | 1.84 | 0.0001 | BB667823 | Rnf125 |
| histocompatibility 2, class II, locus DMa | 1.24 |  | 1.83 | 0.0032 | NM_010386 | H2-DMa |
| macrophage activation 2 like | 1.44 |  | 1.82 | 0.0017 | BG092512 | Mpa2l |
| annexin A5 | 1.11 |  | 1.82 | 0.0001 | D63423 | Anxa5 |
| suppressor of cytokine signaling 5 | 1.28 |  | 1.80 | 0.0006 | AA510713 | Socs5 |
| Fc receptor, IgG, low affinity III | 1.02 |  | 1.75 | 0.0015 | NM_010188 | Fcgr3 |
| insulin-like growth factor binding protein 3 | 1.26 |  | 1.74 | 0.0049 | AV175389 | Igfbp3 |
| serine/threonine kinase 4 | 1.48 | 0.0002 | 1.71 | 0.0005 | BB141897 | Stk4 |
| tissue inhibitor of metalloproteinase 2 | 1.09 |  | 1.68 | 0.0018 | M93954 | Timp2 |
| interferon activated gene 205 /// myeloid cell nuclear differentiation antigen | 1.33 |  | 1.67 | 0.0052 | AI481797 | Ifi205 /// Mnda |
| male sterility domain containing 2 | 1.39 | 0.0004 | 1.67 | 0.0011 | BG094874 | Mlstd2 |
| complement component 9 | 1.42 |  | 1.67 | 0.0065 | NM_013485 | C9 |
| ectonucleoside triphosphate diphosphohydrolase 1 | 1.19 |  | 1.67 | 0.0096 | BI151440 | Entpd1 |
| serum amyloid A 4 | 1.37 |  | 1.66 | 0.0016 | NM_011316 | Saa4 |
| chemokine (C-C motif) ligand 19 | 1.43 |  | 1.64 | 0.0036 | NM_011888 | Ccl19 |
| growth differentiation factor 10 | 1.15 |  | 1.64 | 0.0015 | L42114 | Gdf10 |
| complement component 1, q subcomponent, beta polypeptide | 1.21 |  | 1.62 | 0.0041 | BB111335 | C1qb |
| lymphocyte antigen 6 complex, locus A | 1.35 | 0.0038 | 1.62 | 0.0030 | BC002070 | Ly6a |
| heat shock 70kD protein 5 (glucose-regulated protein) | 1.15 |  | 1.62 | 0.0095 | NM_022310 | Hspa5 |
| cAMP responsive element binding protein 3-like 2 | 1.02 |  | 1.62 | 0.0003 | AV021105 | Creb3l2 |
| E74-like factor 1 | 1.19 |  | 1.61 | 0.0048 | NM_007920 | Elf1 |
| CD86 antigen | 1.32 |  | 1.60 | 0.0012 | NM_019388 | Cd86 |
| vascular endothelial growth factor C | 1.26 | 0.0076 | 1.60 | 0.0032 | AW228853 | Vegfc |
| chemokine (C-C motif) receptor 5 | 1.04 |  | 1.59 | 0.0001 | X94151 | Ccr5 |
| Von Willebrand factor homolog | 1.16 |  | 1.57 | 0.0015 | BB667216 | Vwf |
| PYD and CARD domain containing | 1.27 | 0.0066 | 1.55 | 0.0030 | BG084230 | Pycard |
| serine/threonine kinase 4 | 1.18 |  | 1.53 | 0.0059 | BG069810 | Stk4 |
| tumor necrosis factor (ligand) superfamily, member 12-member 13 | 1.29 |  | 1.52 | 0.0002 | NM_023517 | Tnfsf13 |
| ER degradation enhancer, mannosidase alpha-like 1 | 1.09 |  | 1.51 | 0.0001 | BG064496 | Edem1 |
| ectonucleoside triphosphate diphosphohydrolase 1 | 1.19 |  | 1.51 | 0.0085 | BI151440 | Entpd1 |
| immunity-related GTPase family, Q1 | 1.08 |  | 1.50 | 0.0056 | BM116550 | Irgq1 |
| chemokine (C-C motif) receptor 2 | -1.00 |  | 1.49 | 0.0021 | BB148128 | Ccr2 |
| CD8 antigen, beta chain 1 | 1.06 |  | 1.49 | 0.0059 | BF682469 | Cd8b1 |
| lymphocyte antigen 6 complex, locus E | 1.22 |  | 1.49 | 0.0014 | BM245572 | Ly6e |
| ubiquitin specific peptidase 42 | 1.39 |  | 1.47 | 0.0032 | AW551594 | Usp42 |
| complement component 1, q subcomponent, beta polypeptide | 1.12 |  | 1.45 | 0.0054 | AW227993 | C1qb |
| similar to Interferon-activatable protein 205 (IFI-205) (D3 protein) | 1.23 |  | 1.45 | 0.0016 | M74124 | LOC545386 |
| cold shock domain protein A | 1.27 |  | 1.43 | 0.0048 | BB779100 | Csda |
| 2'-5' oligoadenylate synthetase-like 2 | 1.35 | 0.0074 | 1.42 | 0.0088 | BQ033138 | Oasl2 |
| sequestosome 1 | -1.03 |  | 1.42 | 0.0000 | BM232298 | Sqstm1 |
| Fas apoptotic inhibitory molecule | 1.24 |  | 1.41 | 0.0027 | NM_011810 | Faim |
| cAMP responsive element modulator | 1.01 |  | 1.40 | 0.0038 | AI467599 | Crem |
| general transcription factor II H, polypeptide 2 | 1.03 |  | 1.39 | 0.0058 | NM_022011 | Gtf2h2 |
| retinoblastoma binding protein 8 | 1.04 |  | 1.37 | 0.0093 | BB167067 | Rbbp8 |
| transformation related protein 53 | 1.12 |  | 1.35 | 0.0063 | AJ297973 | Trp53 |
| hemochromatosis | -1.01 |  | 1.35 | 0.0076 | AJ306425 | Hfe |
| fos-like antigen 2 | 1.06 |  | 1.34 | 0.0024 | BM245170 | Fosl2 |
| transformation related protein 53 inducible nuclear protein 1 | -1.07 |  | 1.34 | 0.0025 | AW495711 | Trp53inp1 |
| meningioma expressed antigen 5 (hyaluronidase) | 1.12 |  | 1.34 | 0.0024 | NM_023799 | Mgea5 |
| betacellulin, epidermal growth factor family member | 1.05 |  | 1.33 | 0.0041 | AV231340 | Btc |
| ubiquitin specific peptidase 24 | 1.46 | 0.0006 | 1.32 | 0.0065 | AI196993 | Usp24 |
| haptoglobin | 1.04 |  | 1.31 | 0.0036 | NM_017370 | Hp |
| CCR4-NOT transcription complex, subunit 2 | 1.33 | 0.0050 | 1.30 | 0.0020 | BB220114 | Cnot2 |
| meningioma expressed antigen 5 (hyaluronidase) | 1.08 |  | 1.30 | 0.0040 | AV299787 | Mgea5 |
| general transcription factor II H, polypeptide 4 | 1.22 |  | 1.29 | 0.0013 | NM_010364 | Gtf2h4 |
| cAMP responsive element binding protein 3-like 2 | -1.06 |  | 1.28 | 0.0094 | BB230757 | Creb3l2 |
| phospholipase D family, member 4 | -1.12 |  | 1.27 | 0.0085 | BB210623 | Pld4 |
| heat shock 70kDa protein 14 | 1.13 |  | 1.26 | 0.0021 | NM_015765 | Hspa14 |
| Traf3 interacting protein 2 | -1.01 |  | 1.25 | 0.0045 | NM_134000 | Traf3ip2 |
| Casein kinase 1, delta (Csnk1d), transcript variant 2, mRNA | 1.07 |  | 1.25 | 0.0049 | BB529152 | Csnk1d |
| orosomucoid 1 | 1.16 |  | 1.24 | 0.0044 | BE628912 | Orm1 |
| ligase III, DNA, ATP-dependent | -1.02 |  | 1.23 | 0.0099 | C80352 | Lig3 |
| coagulation factor II (thrombin) receptor | 1.21 | 0.0063 | 1.22 | 0.0044 | AV024285 | F2r |
| similar to interferon-inducible protein 203 | -1.01 |  | 1.21 | 0.0073 | NM_008328 | LOC547362 |
| IK cytokine | 1.11 |  | 1.21 | 0.0037 | AV255179 | Ik |
| heat shock protein 4 | 1.11 |  | 1.18 | 0.0078 | BE912771 | Hspa4 |
| interferon activated gene 204 /// myeloid cell nuclear differentiation antigen | 1.03 |  | 1.17 | 0.0096 | NM_008329 | Ifi204 /// Mnda |
| RAD23b homolog (S. cerevisiae) | 1.15 | 0.0020 | 1.15 | 0.0030 | BF138887 | Rad23b |
| hemopexin | 1.01 |  | 1.15 | 0.0013 | BC011246 | Hpxn |
| retinoblastoma binding protein 4 | 1.20 | 0.0046 | 1.14 | 0.0075 | BB462744 | Rbbp4 |
| retinoblastoma binding protein 7 | 1.14 | 0.0071 | 1.14 | 0.0053 | AV310432 | Rbbp7 |
| SMT3 suppressor of mif two 3 homolog 1 (yeast) | 1.04 |  | -1.17 | 0.0007 | BM209585 | Sumo1 |
| complement component 1, q subcomponent binding protein | -1.04 |  | -1.18 | 0.0012 | NM_007573 | C1qbp |
| insulin-like growth factor binding protein 4 | -1.05 |  | -1.18 | 0.0028 | BB787243 | Igfbp4 |
| insulin-like growth factor binding protein 4 | -1.01 |  | -1.20 | 0.0015 | BB787243 | Igfbp4 |
| kininogen 1 | -1.06 |  | -1.22 | 0.0003 | NM_023125 | Kng1 |
| apurinic/apyrimidinic endonuclease 1 | -1.11 | 0.0064 | -1.27 | 0.0039 | AV263745 | Apex1 |
| Fc receptor, IgG, low affinity IIb | -1.23 | 0.0013 | -1.28 | 0.0004 | BM224327 | Fcgr2b |
| insulin-like growth factor binding protein 4 | -1.10 |  | -1.31 | 0.0000 | BC019836 | Igfbp4 |
| Protein kinase, AMP-activated, alpha 2 catalytic subunit | -1.04 |  | -1.31 | 0.0012 | BQ175911 | Prkaa2 |
| insulin-like growth factor binding protein 4 | -1.09 |  | -1.36 | 0.0001 | NM_010517 | Igfbp4 |
| insulin-like growth factor binding protein 4 | -1.15 |  | -1.36 | 0.0025 | BC019836 | Igfbp4 |
| Fc receptor, IgG, alpha chain transporter | -1.13 |  | -1.38 | 0.0002 | NM_010189 | Fcgrt |
| mitogen activated protein kinase kinase 3 | 1.00 |  | -1.39 | 0.0014 | AI481780 | Map2k3 |
| toll interacting protein | -1.10 |  | -1.40 | 0.0016 | BB400304 | Tollip |
| toll interacting protein | -1.21 | 0.0057 | -1.41 | 0.0051 | BB400304 | Tollip |
| DnaJ (Hsp40) homolog, subfamily B, member 1 | -1.11 |  | -1.41 | 0.0026 | AK002290 | Dnajb1 |
| growth factor receptor bound protein 2-associated protein 1 | -1.14 |  | -1.41 | 0.0061 | NM_021356 | Gab1 |
| chemokine (C-X-C motif) ligand 12 | -1.10 |  | -1.42 | 0.0003 | NM_013655 | Cxcl12 |
| insulin-like growth factor binding protein 4 | -1.04 |  | -1.42 | 0.0047 | NM_010517 | Igfbp4 |
| lysosomal trafficking regulator | -1.10 |  | -1.44 | 0.0033 | BB463428 | Lyst |
| heat shock protein 8 | -1.14 |  | -1.45 | 0.0004 | BC006722 | Hspa8 |
| REV1-like (S. cerevisiae) | -1.08 |  | -1.47 | 0.0056 | NM_019570 | Rev1l |
| growth factor receptor bound protein 2-associated protein 1 | -1.09 |  | -1.48 | 0.0023 | NM_021356 | Gab1 |
| interleukin 17 receptor B | -1.25 |  | -1.49 | 0.0077 | NM_019583 | Il17rb |
| tissue inhibitor of metalloproteinase 3 | -1.04 |  | -1.50 | 0.0039 | BI111620 | Timp3 |
| homocysteine-inducible, endoplasmic reticulum stress-inducible, ubiquitin-like domain member 1 | -1.06 |  | -1.54 | 0.0016 | NM_022331 | Herpud1 |
| fibroblast growth factor receptor-like 1 | -1.18 | 0.0093 | -1.55 | 0.0065 | BB109694 | Fgfrl1 |
| growth factor receptor bound protein 2-associated protein 1 | -1.19 |  | -1.55 | 0.0004 | NM_021356 | Gab1 |
| hepatoma-derived growth factor | -1.12 |  | -1.58 | 0.0006 | C80147 | Hdgf |
| heat shock protein 8 | -1.10 |  | -1.59 | 0.0034 | BC006722 | Hspa8 |
| homocysteine-inducible, endoplasmic reticulum stress-inducible, ubiquitin-like domain member 1 | -1.09 |  | -1.59 | 0.0026 | AI835088 | Herpud1 |
| RAD23a homolog (S. cerevisiae) | -1.25 |  | -1.61 | 0.0072 | NM_009010 | Rad23a |
| fibroblast growth factor 1 | -1.12 |  | -1.63 | 0.0022 | AI649186 | Fgf1 |
| vascular endothelial growth factor A | -1.11 |  | -1.64 | 0.0002 | U50279 | Vegfa |
| low density lipoprotein receptor-related protein associated protein 1 | -1.53 | 0.0041 | -1.65 | 0.0018 | AV309553 | Lrpap1 |
| fibroblast growth factor receptor-like 1 | -1.12 |  | -1.69 | 0.0002 | AF321301 | Fgfrl1 |
| fibroblast growth factor 1 | 1.13 |  | -1.70 | 0.0058 | BE688115 | Fgf1 |
| hepatoma-derived growth factor | -1.26 |  | -1.77 | 0.0012 | NM_008231 | Hdgf |
| vascular endothelial growth factor A | -1.06 |  | -1.78 | 0.0000 | NM_009505 | Vegfa |
| fibroblast growth factor 1 | -1.19 |  | -1.80 | 0.0003 | AI649186 | Fgf1 |
| interleukin 15 receptor, alpha chain | -1.04 |  | -1.83 | 0.0012 | NM_008358 | Il15ra |
| mitogen activated protein kinase kinase 3 | -1.10 |  | -1.83 | 0.0003 | AI481780 | Map2k3 |
| CCAAT/enhancer binding protein (C/EBP), beta | -1.16 |  | -1.85 | 0.0003 | NM_009883 | Cebpb |
| thioredoxin interacting protein | -1.15 |  | -1.91 | 0.0002 | AF173681 | Txnip |
| thioredoxin interacting protein | -1.21 |  | -1.93 | 0.0003 | AF173681 | Txnip |
| CCAAT/enhancer binding protein (C/EBP), beta | -1.24 |  | -2.11 | 0.0004 | AB012278 | Cebpb |
| DNA cross-link repair 1A, PSO2 homolog (S. cerevisiae) | -1.36 |  | -2.43 | 0.0010 | AF241240 | Dclre1a |
| heat shock protein 8 /// similar to heat shock protein 8 /// similar to heat shock protein 8 /// similar to Heat shock cognate 71 kDa protein | -1.29 |  | -2.61 | 0.0026 | AK004608 | Hspa8 |
| CD163 antigen | -2.18 |  | -2.70 | 0.0068 | NM_053094 | Cd163 |
| neuregulin 4 | 1.10 |  | -3.02 | 0.0000 | BB219343 | Nrg4 |
| neuregulin 4 | -1.31 |  | -4.07 | 0.0000 | NM_032002 | Nrg4 |
| hepcidin antimicrobial peptide 1 | -1.23 |  | -5.83 | 0.0059 | NM_032541 | Hamp1 |
| hepcidin antimicrobial peptide 1 | -1.15 |  | -5.86 | 0.0067 | NM_032541 | Hamp1 |
| ubiquitin specific protease 2 | -1.18 |  | -9.73 | 0.0000 | AI553394 | Usp2 |
| ubiquitin specific protease 2 | -1.22 | 0.0053 | -11.75 | 0.0000 | AI553394 | Usp2 |
