# Supplementary material for: Atherosclerosis and liver inflammation induced by increased dietary cholesterol intake: a combined transcriptomics and metabolomics analysis
Source: Genome Biol. 2007 Sep 24;8(9):R200. doi: 10.1186/gb-2007-8-9-r200 (PMC2375038; doi:10.1186/gb-2007-8-9-r200)
Supplement: Additional data file 4 — Canonical pathway analysis for cholesterol metabolism and analysis of the gene expression data based on GO annotation with disease categories (MetaCore™ software, GeneGO). [file gb-2007-8-9-r200-S4.ppt]

## Slide 1
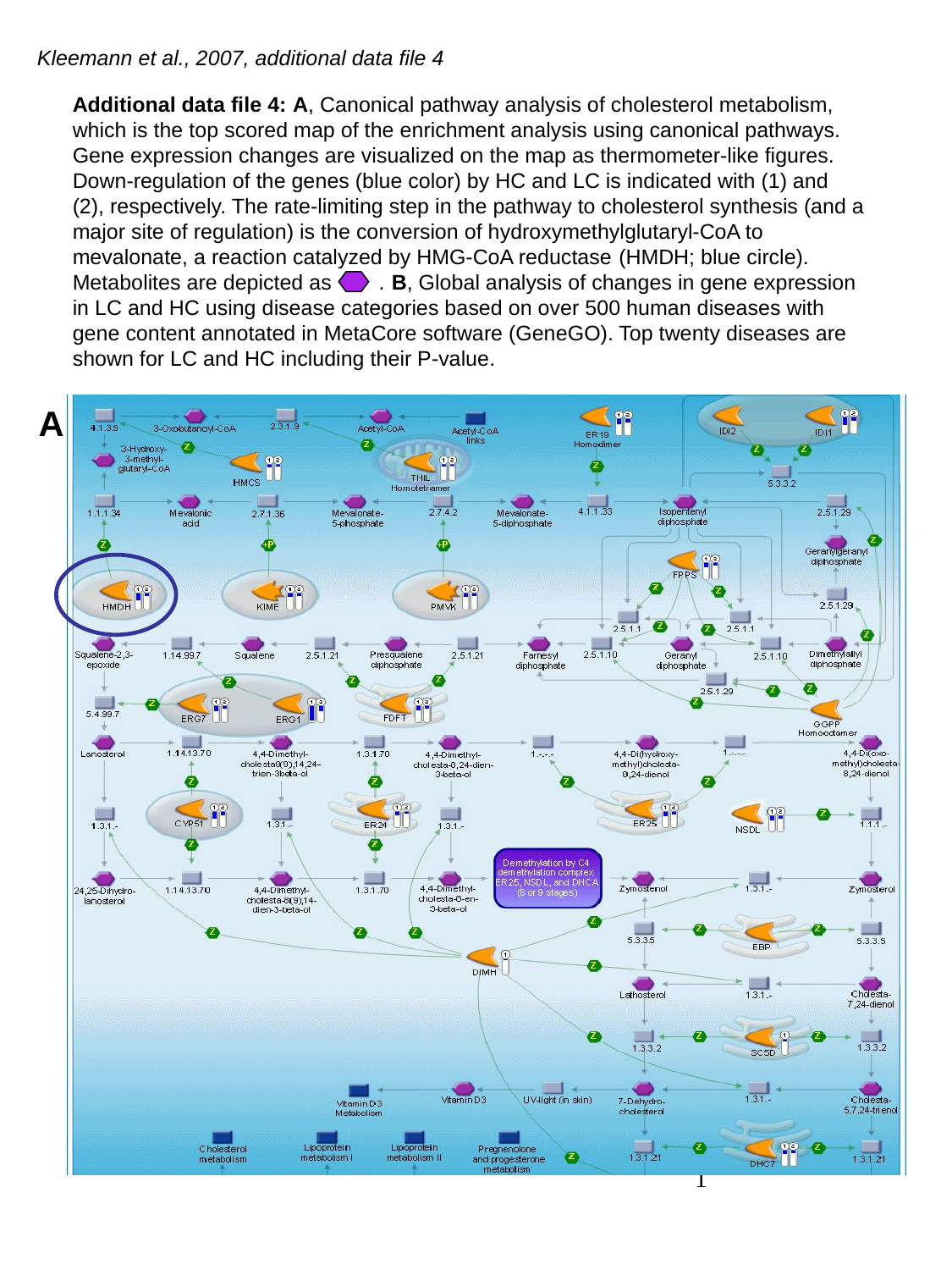

Kleemann et al., 2007, additional data file 4
Additional data file 4: A, Canonical pathway analysis of cholesterol metabolism, which is the top scored map of the enrichment analysis using canonical pathways. Gene expression changes are visualized on the map as thermometer-like figures. Down-regulation of the genes (blue color) by HC and LC is indicated with (1) and (2), respectively. The rate-limiting step in the pathway to cholesterol synthesis (and a major site of regulation) is the conversion of hydroxymethylglutaryl-CoA to mevalonate, a reaction catalyzed by HMG-CoA reductase (HMDH; blue circle). Metabolites are depicted as . B, Global analysis of changes in gene expression in LC and HC using disease categories based on over 500 human diseases with gene content annotated in MetaCore software (GeneGO). Top twenty diseases are shown for LC and HC including their P-value.
A
1

## Slide 2
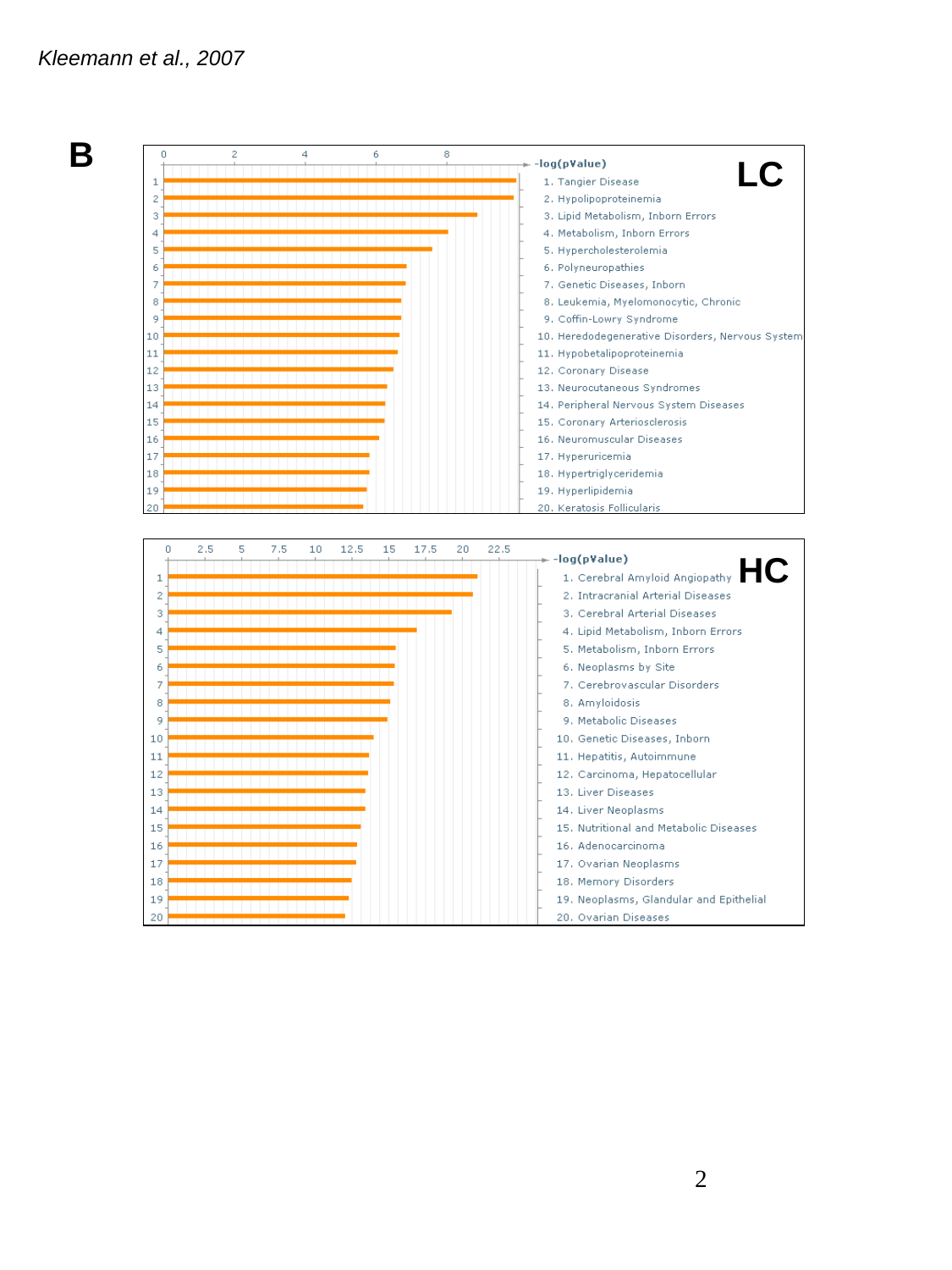

Kleemann et al., 2007
B
LC
HC
2
