# Supplementary material for: Atherosclerosis and liver inflammation induced by increased dietary cholesterol intake: a combined transcriptomics and metabolomics analysis
Source: Genome Biol. 2007 Sep 24;8(9):R200. doi: 10.1186/gb-2007-8-9-r200 (PMC2375038; doi:10.1186/gb-2007-8-9-r200)
Supplement: Additional data file 5 — Comprehensive network analysis (functional OMICs analysis) by merging gene expression datasets with the metabolite datasets using MetaCore™ network software. [file gb-2007-8-9-r200-S5.ppt]

## Slide 1
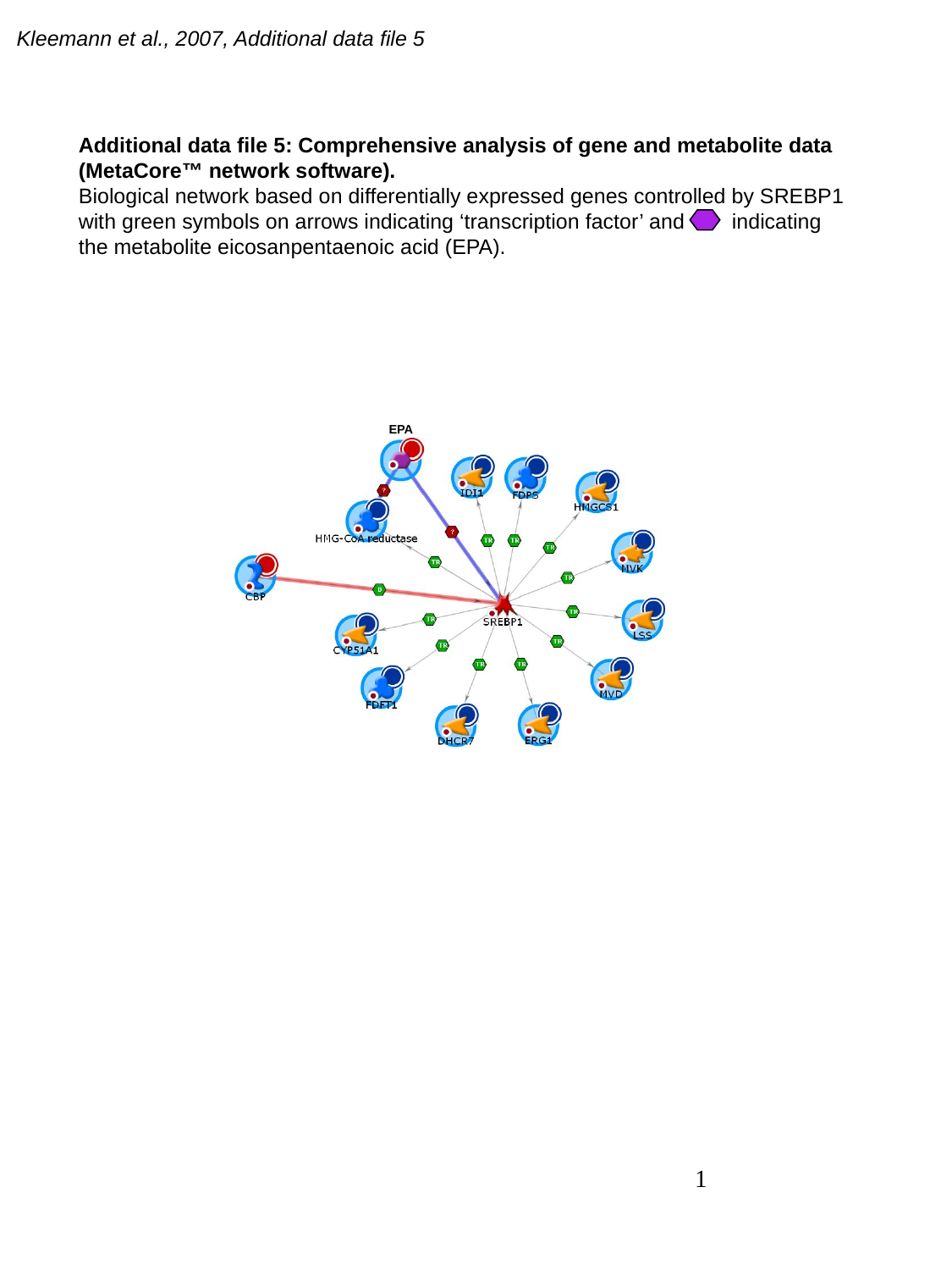

Kleemann et al., 2007, Additional data file 5
Additional data file 5: Comprehensive analysis of gene and metabolite data (MetaCore™ network software).
Biological network based on differentially expressed genes controlled by SREBP1 with green symbols on arrows indicating ‘transcription factor’ and indicating the metabolite eicosanpentaenoic acid (EPA).
EPA
1
