# Supplementary material for: Atherosclerosis and liver inflammation induced by increased dietary cholesterol intake: a combined transcriptomics and metabolomics analysis
Source: Genome Biol. 2007 Sep 24;8(9):R200. doi: 10.1186/gb-2007-8-9-r200 (PMC2375038; doi:10.1186/gb-2007-8-9-r200)
Supplement: Additional data file 6 — Biological networks of differentially expressed genes in the HC group allowing the identification of transcriptional master regulators. [file gb-2007-8-9-r200-S6.ppt]

## Slide 1
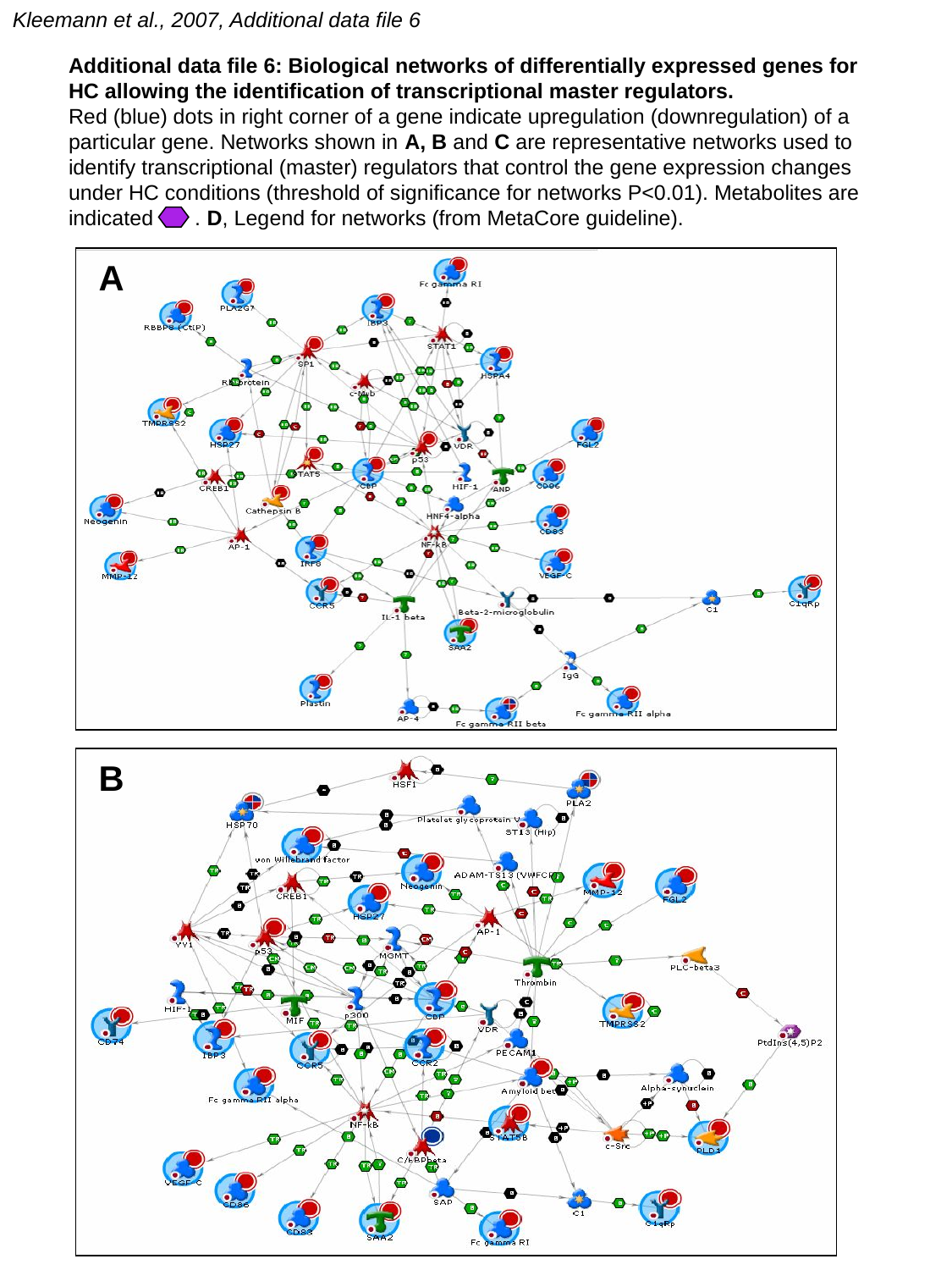

Kleemann et al., 2007, Additional data file 6
Additional data file 6: Biological networks of differentially expressed genes for HC allowing the identification of transcriptional master regulators.
Red (blue) dots in right corner of a gene indicate upregulation (downregulation) of a particular gene. Networks shown in A, B and C are representative networks used to identify transcriptional (master) regulators that control the gene expression changes under HC conditions (threshold of significance for networks P<0.01). Metabolites are indicated . D, Legend for networks (from MetaCore guideline).
A
B
1

## Slide 2
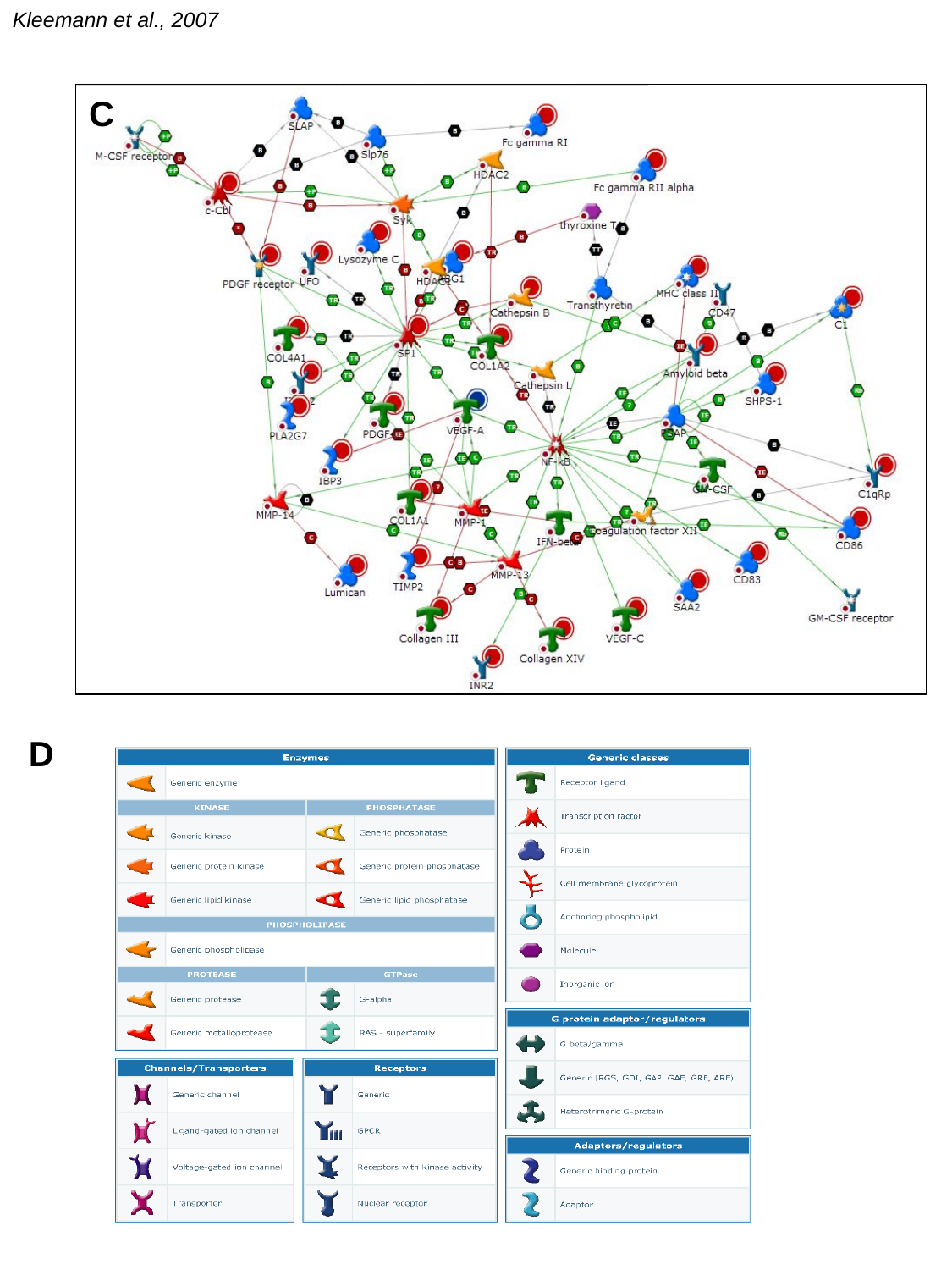

Kleemann et al., 2007
C
D
2

## Slide 3
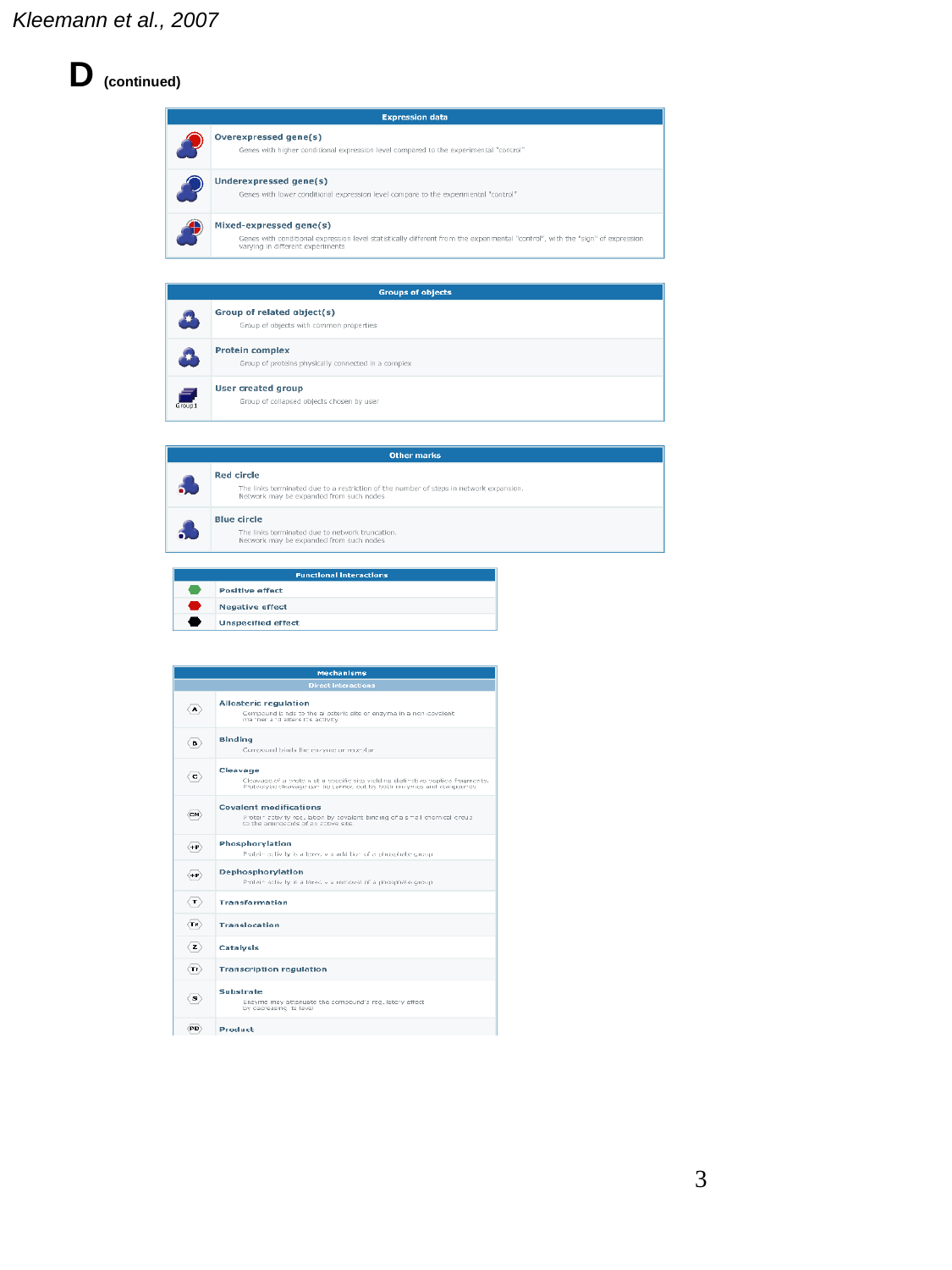

Kleemann et al., 2007
D (continued)
3
