# Supplementary material for: Atherosclerosis and liver inflammation induced by increased dietary cholesterol intake: a combined transcriptomics and metabolomics analysis
Source: Genome Biol. 2007 Sep 24;8(9):R200. doi: 10.1186/gb-2007-8-9-r200 (PMC2375038; doi:10.1186/gb-2007-8-9-r200)
Supplement: Additional data file 7 — Cholesterol-induced factors with reported extracellular function. [file gb-2007-8-9-r200-S7.doc]

**Additional data file 7:** **Cholesterol-induced factors with extracellular function.** Cholesterol-inducible hepatic factors with putative pro-atherogenic effects after secretion by the liver were identified based on the GO selection criteria ‘cellular component: extracellular’ and data mining. Genes of which gene products have been reported to be pro-atherogenic are highlighted blue. The inducing effect of LC and HC treatment is indicated as a fold change compared to Con and listed together with a P-value. Significant up-regulation of P<0.01 is indicated in red.

|  | **LC** | | **HC** | |  |  |
| --- | --- | --- | --- | --- | --- | --- |
| **Gene name** | **Fold change** | **P-value** | **Fold change** | **P-value** | **GenBank-id number** | **Gene symbol** |
| glycoprotein (transmembrane) nmb | 1.59 |  | 14.87 | 0.0000 | NM_053110 | Gpnmb |
| lipocalin 2 | 1.41 |  | 5.90 | 0.0010 | X14607 | Lcn2 |
| **matrix metalloproteinase 12** | 1.77 |  | 5.68 | 0.0028 | BC019135 | Mmp12 |
| **lipoprotein lipase** | 1.83 | 0.0030 | 5.47 | 0.0000 | AK017272 | Lpl |
| claudin 1 | 1.35 |  | 4.19 | 0.0000 | NM_016674 | Cldn1 |
| serum amyloid A 3 | 1.45 |  | 4.11 | 0.0002 | NM_011315 | Saa3 |
| orosomucoid 2 | 1.76 | 0.0060 | 4.05 | 0.0001 | NM_011016 | Orm2 |
| claudin 1 | 1.47 | 0.0051 | 3.88 | 0.0000 | AV227581 | Cldn1 |
| serum amyloid A 1 | 1.48 |  | 3.55 | 0.0029 | NM_009117 | Saa1 |
| serum amyloid A 1 | 1.48 |  | 3.45 | 0.0013 | NM_011314 | Saa1 |
| phospholipase A2, group VII (platelet-activating factor acetylhydrolase, plasma) | 1.57 |  | 3.37 | 0.0001 | AK005158 | Pla2g7 |
| serum amyloid A 2 | 1.39 |  | 3.24 | 0.0025 | NM_011314 | Saa2 |
| procollagen, type III, alpha 1 | 1.36 |  | 3.18 | 0.0037 | AW550625 | Col3a1 |
| carbonic anhydrase 14 | 1.36 |  | 3.10 | 0.0001 | NM_011797 | Car14 |
| CD83 antigen | 1.66 |  | 3.03 | 0.0007 | NM_009856 | Cd83 |
| histocompatibility 2, class II antigen E beta | 1.62 |  | 2.93 | 0.0000 | NM_010382 | H2-Eb1 |
| insulin-like growth factor binding protein 5 | 1.51 |  | 2.81 | 0.0001 | BF225802 | Igfbp5 |
| eosinophil-associated, ribonuclease A family, member 1 /// member 2 /// member 3 /// member 12 | 1.63 | 0.0038 | 2.80 | 0.0000 | NM_017388 | Ear1; -2; Ear3; -12 |
| cytochrome P450, family 2, subfamily c, polypeptide 55 | 1.00 |  | 2.73 | 0.0049 | NM_028089 | Cyp2c55 |
| chemokine (C-C motif) ligand 6 | 1.24 |  | 2.67 | 0.0001 | BC002073 | Ccl6 |
| procollagen, type I, alpha 2 | 1.14 |  | 2.59 | 0.0024 | BF227507 | Col1a2 |
| chemokine (C-C motif) ligand 6 | 1.21 |  | 2.52 | 0.0002 | AV084904 | Ccl6 |
| fibrinogen-like protein 2 | 1.78 | 0.0008 | 2.51 | 0.0003 | BF136544 | Fgl2 |
| follistatin-like 1 | 1.55 |  | 2.48 | 0.0001 | BI452727 | Fstl1 |
| P lysozyme structural | 1.36 |  | 2.46 | 0.0000 | AV066625 | Lzp-s |
| lysozyme | 1.35 |  | 2.45 | 0.0001 | AW208566 | Lyzs |
| procollagen, type I, alpha 1 | 1.22 |  | 2.41 | 0.0025 | U08020 | Col1a1 |
| procollagen, type I, alpha 2 | 1.17 |  | 2.34 | 0.0009 | BF227507 | Col1a2 |
| ecotropic viral integration site 2a | 1.43 |  | 2.31 | 0.0002 | NM_010161 | Evi2a |
| protease, serine, 23 | 1.35 |  | 2.29 | 0.0013 | BB378796 | Prss23 |
| procollagen, type V, alpha 2 | 1.24 |  | 2.29 | 0.0010 | AV229424 | Col5a2 |
| P lysozyme structural | 1.27 |  | 2.27 | 0.0000 | AV058500 | Lzp-s |
| lymphocyte antigen 86 | 1.23 |  | 2.26 | 0.0000 | NM_010745 | Ly86 |
| protein tyrosine phosphatase, non-receptor type substrate 1 | 1.16 |  | 2.22 | 0.0015 | AB018194 | Ptpns1 |
| serine (or cysteine) proteinase inhibitor, clade E, member 2 | 1.49 |  | 2.19 | 0.0015 | NM_009255 | Serpine2 |
| leptin receptor overlapping transcript-like 1 | 1.46 | 0.0034 | 2.19 | 0.0000 | BF658789 | Leprotl1 |
| procollagen, type VI, alpha 3 | 1.38 |  | 2.18 | 0.0020 | AF064749 | Col6a3 |
| CD5 antigen-like | 1.33 |  | 2.18 | 0.0001 | NM_009690 | Cd5l |
| GLI pathogenesis-related 1 (glioma) | 1.26 |  | 2.17 | 0.0022 | BC025083 | Glipr1 |
| platelet-derived growth factor, C polypeptide | 1.02 |  | 2.16 | 0.0000 | NM_019971 | Pdgfc |
| cathepsin S | 1.19 |  | 2.16 | 0.0001 | NM_021281 | Ctss |
| TPA regulated locus | 1.25 | 0.0086 | 2.12 | 0.0000 | BQ173970 | Tparl |
| platelet derived growth factor receptor, beta polypeptide | 1.40 | 0.0024 | 2.10 | 0.0000 | AA499047 | Pdgfrb |
| latent transforming growth factor beta binding protein 3 | 1.56 | 0.0028 | 2.05 | 0.0044 | BB324823 | Ltbp3 |
| ATP-binding cassette, sub-family G (WHITE), member 1 | 1.35 |  | 2.05 | 0.0008 | AW413978 | Abcg1 |
| junction adhesion molecule 2 | 1.55 |  | 2.03 | 0.0008 | AU016127 | Jam2 |
| CD68 antigen | 1.05 |  | 2.03 | 0.0010 | BC021637 | Cd68 |
| cysteine rich transmembrane BMP regulator 1 (chordin like) | 1.72 |  | 2.02 | 0.0028 | AK018666 | Crim1 |
| complement component 1, q subcomponent, receptor 1 | 1.02 |  | 2.00 | 0.0022 | AV319144 | C1qr1 |
| integrin beta 2 | 1.27 |  | 2.00 | 0.0001 | NM_008404 | Itgb2 |
| follistatin-like 1 | 1.12 |  | 1.99 | 0.0087 | BI452727 | Fstl1 |
| procollagen, type IV, alpha 1 | 1.29 |  | 1.98 | 0.0035 | BF158638 | Col4a1 |
| adrenomedullin | 1.34 |  | 1.97 | 0.0004 | NM_009627 | Adm |
| protein tyrosine phosphatase, non-receptor type substrate 1 | 1.22 |  | 1.95 | 0.0006 | AB018194 | Ptpns1 |
| nidogen 1 | 1.31 |  | 1.94 | 0.0012 | X14480 | Nid1 |
| solute carrier family 13 (sodium-dependent dicarboxylate transporter), member 3 | 1.34 | 0.0047 | 1.94 | 0.0000 | BB497312 | Slc13a3 |
| solute carrier family 13 (sodium-dependent dicarboxylate transporter), member 3 | 1.39 | 0.0052 | 1.91 | 0.0022 | NM_054055 | Slc13a3 |
| interleukin 18 binding protein | 1.13 |  | 1.89 | 0.0007 | AF110803 | Il18bp |
| lumican | 1.11 |  | 1.87 | 0.0014 | AK014312 | Lum |
| Fc receptor, IgG, high affinity I | 1.47 |  | 1.85 | 0.0008 | AF143181 | Fcgr1 |
| glycoprotein 49 A /// leukocyte immunoglobulin-like receptor, subfamily B, member 4 | 1.29 |  | 1.85 | 0.0035 | U05264 | Gp49a /// Lilrb4 |
| matrix metalloproteinase 19 | 1.27 |  | 1.84 | 0.0007 | AF153199 | Mmp19 |
| armadillo repeat containing, X-linked 3 | 1.27 |  | 1.81 | 0.0004 | AK004598 | Armcx3 |
| protease, serine, 23 | 1.14 | 0.0069 | 1.80 | 0.0002 | AK009847 | Prss23 |
| platelet derived growth factor receptor, alpha polypeptide | 1.36 |  | 1.80 | 0.0008 | AW537708 | Pdgfra |
| TYRO protein tyrosine kinase binding protein | 1.02 |  | 1.80 | 0.0012 | NM_011662 | Tyrobp |
| cathepsin B | 1.10 |  | 1.78 | 0.0000 | M14222 | Ctsb |
| Fc receptor, IgG, low affinity III | 1.02 |  | 1.75 | 0.0015 | NM_010188 | Fcgr3 |
| insulin-like growth factor binding protein 3 | 1.26 |  | 1.74 | 0.0049 | AV175389 | Igfbp3 |
| epoxide hydrolase 1, microsomal | -1.01 |  | 1.74 | 0.0002 | NM_010145 | Ephx1 |
| syndecan 1 | 1.01 |  | 1.73 | 0.0002 | BI788645 | Sdc1 |
| procollagen, type XIV, alpha 1 | 1.24 |  | 1.70 | 0.0006 | AJ131395 | Col14a1 |
| thrombospondin 2 | 1.09 |  | 1.70 | 0.0012 | NM_011581 | Thbs2 |
| cathepsin L | 1.30 |  | 1.69 | 0.0027 | AV023994 | Ctsl |
| tissue inhibitor of metalloproteinase 2 | 1.09 |  | 1.68 | 0.0018 | M93954 | Timp2 |
| syndecan 1 | 1.20 |  | 1.68 | 0.0003 | BB533095 | Sdc1 |
| complement component 9 | 1.42 |  | 1.67 | 0.0065 | NM_013485 | C9 |
| protein tyrosine phosphatase, receptor type, K | 1.28 |  | 1.66 | 0.0030 | AI893646 | Ptprk |
| serum amyloid A 4 | 1.37 |  | 1.66 | 0.0016 | NM_011316 | Saa4 |
| AXL receptor tyrosine kinase | 1.06 |  | 1.65 | 0.0024 | AA500897 | Axl |
| transmembrane emp24 domain containing 3 | 1.20 |  | 1.65 | 0.0011 | NM_025360 | Tmed3 |
| Prolyl 4-hydroxylase, beta polypeptide | 1.06 |  | 1.65 | 0.0039 | BB414484 | P4hb |
| chemokine (C-C motif) ligand 19 | 1.43 |  | 1.64 | 0.0036 | NM_011888 | Ccl19 |
| growth differentiation factor 10 | 1.15 |  | 1.64 | 0.0015 | L42114 | Gdf10 |
| interferon (alpha and beta) receptor 2 | 1.10 |  | 1.64 | 0.0084 | BB522265 | Ifnar2 |
| stress 70 protein chaperone, microsome-associated, human homolog | 1.32 | 0.0003 | 1.63 | 0.0002 | BE650268 | Stch |
| complement component 1, q subcomponent, beta polypeptide | 1.21 |  | 1.62 | 0.0041 | BB111335 | C1qb |
| galactosidase, alpha | 1.31 |  | 1.62 | 0.0057 | NM_013463 | Gla |
| frizzled-related protein | 1.12 | 0.0093 | 1.62 | 0.0022 | U91905 | Frzb |
| leptin receptor overlapping transcript-like 1 | 1.19 |  | 1.61 | 0.0002 | BF658789 | Leprotl1 |
| vascular endothelial growth factor C | 1.26 | 0.0076 | 1.60 | 0.0032 | AW228853 | Vegfc |
| jagged 1 | 1.38 |  | 1.60 | 0.0075 | AV359819 | Jag1 |
| murinoglobulin 1 | 1.52 | 0.0004 | 1.59 | 0.0006 | NM_008645 | Mug1 |
| procollagen, type V, alpha 1 | 1.06 |  | 1.58 | 0.0035 | AW744319 | Col5a1 |
| a disintegrin-like and metalloprotease (reprolysin type) with thrombospondin type 1 motif, 5 (aggrecanase-2) | 1.03 |  | 1.57 | 0.0048 | BB658835 | Adamts5 |
| Exostoses (multiple) 1 | 1.30 |  | 1.57 | 0.0034 | BM231698 | Ext1 |
| Von Willebrand factor homolog | 1.16 |  | 1.57 | 0.0015 | BB667216 | Vwf |
| cathepsin B | 1.03 |  | 1.57 | 0.0086 | M14222 | Ctsb |
| platelet-derived growth factor, C polypeptide | -1.16 |  | 1.57 | 0.0008 | NM_019971 | Pdgfc |
| epidermal growth factor-containing fibulin-like extracellular matrix protein 2 | 1.22 |  | 1.57 | 0.0034 | NM_021474 | Efemp2 |
| Colony stimulating factor 2 receptor, beta 1, low-affinity (granulocyte-macrophage) | 1.08 |  | 1.56 | 0.0058 | BB769628 | Csf2rb1 |
| S100 calcium binding protein A11 (calizzarin) | 1.14 |  | 1.56 | 0.0042 | BC021916 | S100a11 |
| endothelin receptor type B | 1.16 |  | 1.56 | 0.0015 | BF100813 | Ednrb |
| procollagen C-proteinase enhancer protein | 1.23 |  | 1.56 | 0.0021 | BB250811 | Pcolce |
| syndecan 1 | 1.00 |  | 1.56 | 0.0013 | BI788645 | Sdc1 |
| stress 70 protein chaperone, microsome-associated, human homolog | 1.13 |  | 1.56 | 0.0014 | BE533039 | Stch |
| proteoglycan 1, secretory granule | 1.15 |  | 1.56 | 0.0001 | NM_011157 | Prg1 |
| PYD and CARD domain containing | 1.27 | 0.0066 | 1.55 | 0.0030 | BG084230 | Pycard |
| UBX domain containing 2 | 1.02 |  | 1.53 | 0.0014 | AI788596 | Ubxd2 |
| claudin 2 | 1.04 |  | 1.53 | 0.0000 | NM_016675 | Cldn2 |
| claudin 2 | 1.14 |  | 1.52 | 0.0006 | NM_016675 | Cldn2 |
| tumor necrosis factor (ligand) superfamily, member 13 | 1.29 |  | 1.52 | 0.0002 | NM_023517 | Tnfsf13 |
| biglycan | 1.36 |  | 1.51 | 0.0087 | BC019502 | Bgn |
| transmembrane emp24 protein transport domain containing 9 | 1.11 |  | 1.51 | 0.0007 | NM_026211 | Tmed9 |
| biglycan | 1.20 |  | 1.50 | 0.0041 | BC019502 | Bgn |
| procollagen, type XIV, alpha 1 | 1.31 |  | 1.49 | 0.0006 | BB521934 | Col14a1 |
| lymphocyte antigen 6 complex, locus E | 1.22 |  | 1.49 | 0.0014 | BM245572 | Ly6e |
| laminin B1 subunit 1 | 1.03 |  | 1.48 | 0.0001 | BG970109 | Lamb1-1 |
| integrin beta 5 | 1.10 |  | 1.47 | 0.0001 | BB543646 | Itgb5 |
| NADPH oxidase 4 | 1.73 | 0.0005 | 1.47 | 0.0087 | BC021378 | Nox4 |
| insulin-like growth factor binding protein 7 | 1.34 |  | 1.47 | 0.0082 | AI481026 | Igfbp7 |
| complement component 1, q subcomponent, beta polypeptide | 1.12 |  | 1.45 | 0.0054 | AW227993 | C1qb |
| FXYD domain-containing ion transport regulator 5 | 1.13 |  | 1.45 | 0.0078 | NM_008761 | Fxyd5 |
| hyaluronan and proteoglycan link protein 4 | 1.12 |  | 1.44 | 0.0076 | BB082407 | Hapln4 |
| cathepsin B | 1.09 |  | 1.43 | 0.0000 | M14222 | Ctsb |
| Exostoses (multiple) 1 | 1.08 |  | 1.42 | 0.0030 | BB497623 | Ext1 |
| stabilin 2 | 1.09 |  | 1.41 | 0.0000 | NM_138673 | Stab2 |
| oncostatin M receptor | 1.12 |  | 1.40 | 0.0062 | AB015978 | Osmr |
| UBX domain containing 2 | 1.12 |  | 1.39 | 0.0001 | AI788596 | Ubxd2 |
| cathepsin L | 1.13 |  | 1.39 | 0.0001 | J02583 | Ctsl |
| fibrillin 1 | 1.15 |  | 1.39 | 0.0035 | NM_007993 | Fbn1 |
| CD164 antigen | 1.23 |  | 1.39 | 0.0008 | NM_016898 | Cd164 |
| pituitary tumor-transforming 1 interacting protein | 1.30 | 0.0015 | 1.38 | 0.0019 | AU018448 | Pttg1ip |
| protein tyrosine phosphatase, receptor type, F | 1.13 |  | 1.38 | 0.0005 | BF235516 | Ptprf |
| integrin beta 5 | 1.09 |  | 1.38 | 0.0023 | BB543979 | Itgb5 |
| cathepsin B | 1.08 |  | 1.38 | 0.0000 | M14222 | Ctsb |
| UBX domain containing 2 | 1.03 |  | 1.37 | 0.0002 | AI788596 | Ubxd2 |
| prosaposin | -1.02 |  | 1.37 | 0.0003 | BM212050 | Psap |
| prosaposin | 1.01 |  | 1.37 | 0.0002 | BM212050 | Psap |
| coxsackievirus and adenovirus receptor | 1.22 |  | 1.36 | 0.0016 | BE824924 | Cxadr |
| reticulocalbin 1 | 1.26 | 0.0001 | 1.36 | 0.0002 | NM_009037 | Rcn1 |
| netrin 1 | 1.29 |  | 1.35 | 0.0063 | BI143915 | Ntn1 |
| protein-tyrosine sulfotransferase 2 | 1.20 |  | 1.35 | 0.0064 | NM_009419 | Tpst2 |
| hemochromatosis | -1.01 |  | 1.35 | 0.0076 | AJ306425 | Hfe |
| betacellulin, epidermal growth factor family member | 1.05 |  | 1.33 | 0.0041 | AV231340 | Btc |
| cathepsin Z | 1.08 |  | 1.33 | 0.0004 | NM_022325 | Ctsz |
| TPA regulated locus | 1.03 |  | 1.33 | 0.0016 | NM_011626 | Tparl |
| carboxypeptidase B2 (plasma) | 1.23 | 0.0065 | 1.33 | 0.0004 | NM_019775 | Cpb2 |
| folylpolyglutamyl synthetase | -1.13 |  | 1.33 | 0.0088 | U33557 | Fpgs |
| FK506 binding protein 7 | 1.14 |  | 1.32 | 0.0047 | NM_010222 | Fkbp7 |
| FK506 binding protein 9 | 1.11 |  | 1.32 | 0.0025 | BB026630 | Fkbp9 |
| a disintegrin and metalloproteinase domain 17 | 1.07 |  | 1.31 | 0.0056 | C76813 | Adam17 |
| haptoglobin | 1.04 |  | 1.31 | 0.0036 | NM_017370 | Hp |
| integrin beta 5 | -1.01 |  | 1.31 | 0.0021 | NM_010580 | Itgb5 |
| phospholipase A2, group XIIB | 1.05 |  | 1.31 | 0.0004 | BC021592 | Pla2g12b |
| integrin beta 5 | -1.00 |  | 1.31 | 0.0010 | NM_010580 | Itgb5 |
| dehydrogenase/reductase (SDR family) member 3 | 1.10 |  | 1.31 | 0.0069 | NM_011303 | Dhrs3 |
| tuftelin interacting protein 11 | 1.26 |  | 1.30 | 0.0068 | NM_018783 | Tfip11 |
| SM-11044 binding protein | 1.07 |  | 1.30 | 0.0093 | AK004283 | MGI:1914262 |
| bisphosphate 3'-nucleotidase 1 | 1.24 | 0.0081 | 1.30 | 0.0032 | BB412311 | Bpnt1 |
| camello-like 2 | 1.34 | 0.0087 | 1.29 | 0.0024 | BB745660 | Cml2 |
| solute carrier family 39 (zinc transporter), member 7 | 1.06 |  | 1.28 | 0.0081 | NM_008202 | Slc39a7 |
| cathepsin Z | 1.11 |  | 1.28 | 0.0058 | NM_022325 | Ctsz |
| angiopoietin-like 3 | 1.03 |  | 1.28 | 0.0009 | BC019491 | Angptl3 |
| sulfatase 2 | 1.08 |  | 1.27 | 0.0016 | AU020235 | Sulf2 |
| insulin degrading enzyme | 1.06 |  | 1.27 | 0.0002 | AK004972 | Ide |
| cathepsin Z | -1.00 |  | 1.27 | 0.0027 | NM_022325 | Ctsz |
| pituitary tumor-transforming 1 interacting protein | 1.05 |  | 1.27 | 0.0000 | BB498753 | Pttg1ip |
| protein tyrosine phosphatase, receptor type, F | 1.19 |  | 1.26 | 0.0020 | BF235516 | Ptprf |
| intergral membrane protein 1 | 1.15 |  | 1.26 | 0.0042 | AA756810 | Itm1 |
| ectonucleotide pyrophosphatase/phosphodiesterase 5 | 1.16 | 0.0047 | 1.26 | 0.0021 | BC011294 | Enpp5 |
| mitochondrial ribosomal protein L36 | 1.10 |  | 1.24 | 0.0046 | AB049655 | Mrpl36 |
| orosomucoid 1 | 1.16 |  | 1.24 | 0.0044 | BE628912 | Orm1 |
| solute carrier family 25 (mitochondrial carrier, brain), member 14 | 1.00 |  | 1.23 | 0.0069 | NM_011398 | Slc25a14 |
| thymic stromal-derived lymphopoietin, receptor | 1.12 | 0.0052 | 1.23 | 0.0003 | NM_016715 | Tslpr |
| plexin C1 | 1.03 |  | 1.22 | 0.0073 | BB476707 | Plxnc1 |
| ceroid-lipofuscinosis, neuronal 2 | 1.14 | 0.0053 | 1.22 | 0.0002 | NM_009906 | Cln2 |
| coagulation factor II (thrombin) receptor | 1.21 | 0.0063 | 1.22 | 0.0044 | AV024285 | F2r |
| clusterin | 1.03 |  | 1.22 | 0.0024 | BB433678 | Clu |
| spastic paraplegia 20, spartin (Troyer syndrome) homolog (human) | 1.23 | 0.0017 | 1.22 | 0.0022 | BB040507 | Spg20 |
| granulin | 1.01 |  | 1.19 | 0.0041 | AV166504 | Grn |
| multiple coagulation factor deficiency 2 | -1.05 |  | 1.17 | 0.0034 | BC003996 | Mcfd2 |
| ATPase, H+ transporting, lysosomal accessory protein 1 | 1.04 |  | 1.16 | 0.0086 | AI316502 | Atp6ap1 |
| apolipoprotein E | 1.04 |  | 1.15 | 0.0009 | AK019319 | Apoe |
| hemopexin | 1.01 |  | 1.15 | 0.0013 | BC011246 | Hpxn |
| SM-11044 binding protein | 1.10 |  | 1.12 | 0.0051 | AV061337 | MGI:1914262 |
| alpha 1 microglobulin/bikunin | 1.06 |  | 1.09 | 0.0008 | NM_007443 | Ambp |
| transferrin | -1.01 |  | 1.07 | 0.0015 | AF440692 | Trf |
