# Supplementary material for: Atherosclerosis and liver inflammation induced by increased dietary cholesterol intake: a combined transcriptomics and metabolomics analysis
Source: Genome Biol. 2007 Sep 24;8(9):R200. doi: 10.1186/gb-2007-8-9-r200 (PMC2375038; doi:10.1186/gb-2007-8-9-r200)
Supplement: Additional data file 8 — Quality control analysis steps for RNA samples prior to hybridization on Affymetrix microarrays usig Agilent Lab-on-a-chip technology. [file gb-2007-8-9-r200-S8.ppt]

## Slide 1
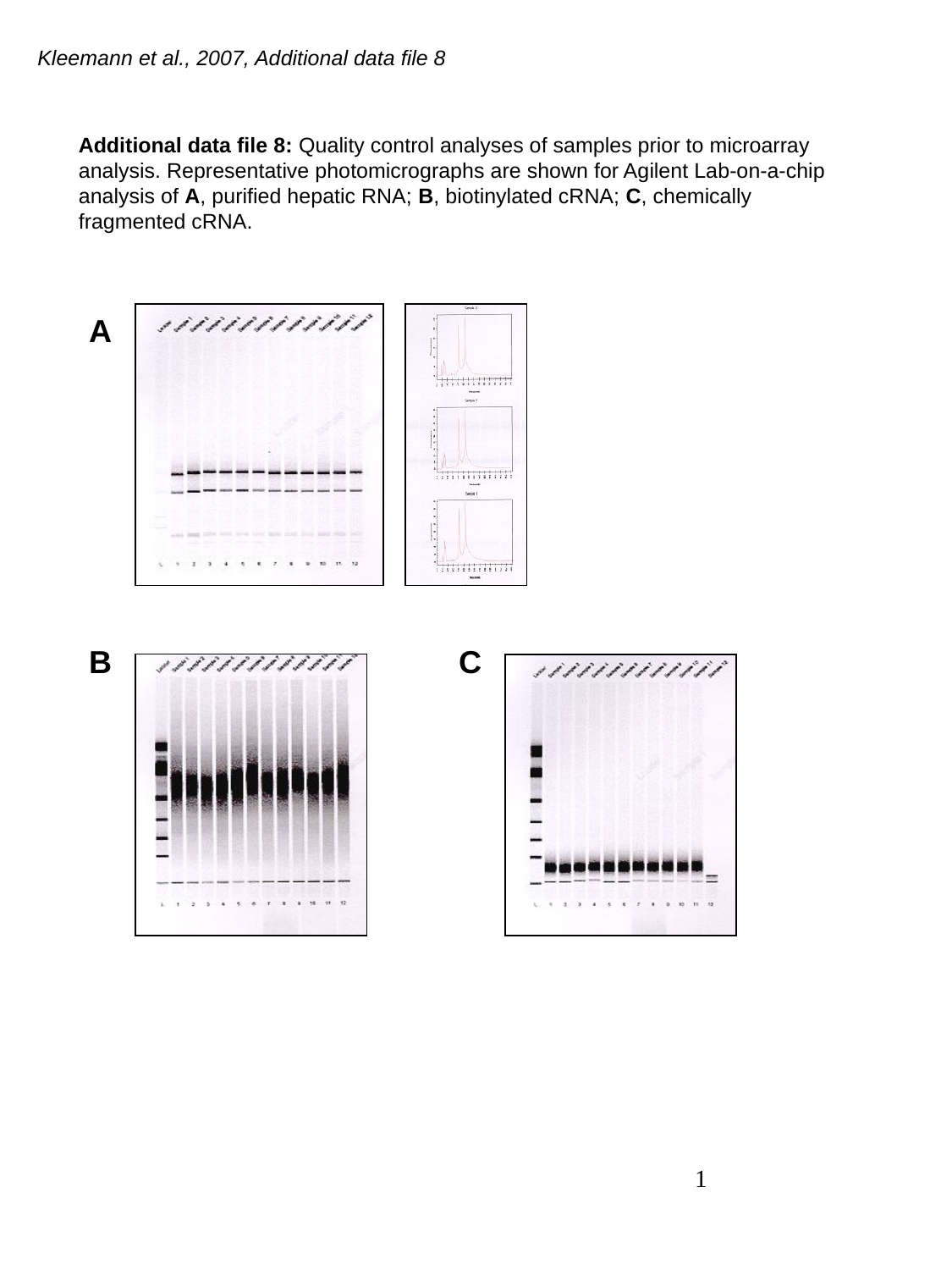

Kleemann et al., 2007, Additional data file 8
Additional data file 8: Quality control analyses of samples prior to microarray analysis. Representative photomicrographs are shown for Agilent Lab-on-a-chip analysis of A, purified hepatic RNA; B, biotinylated cRNA; C, chemically fragmented cRNA.
A
B
C
1
